# Lipidomic Profile Revealed the Association of Plasma Lysophosphatidylcholines with Adolescent Obesity

**DOI:** 10.1155/2019/1382418

**Published:** 2019-12-13

**Authors:** Yang Wang, Chang-Tao Jiang, Jie-Yun Song, Qi-Ying Song, Jun Ma, Hai-Jun Wang

**Affiliations:** ^1^Department of Maternal and Child Health, School of Public Health, Peking University, Beijing 100191, China; ^2^Department of Physiology and Pathophysiology, School of Basic Medical Sciences, Peking University, Beijing 100191, China; ^3^Institute of Child and Adolescent Health, Peking University, Beijing 100191, China

## Abstract

**Objective:**

The human lipidomic profile reflects lipid metabolism, including the early phase of pathophysiological changes associated with diseases. An investigation of the association between the plasma lipidomic profile and adolescent obesity might provide new insights into the biological mechanisms of obesity. Therefore, we aimed to investigate the association of the plasma lipidome with obesity in Chinese adolescents using lipidomics.

**Methods:**

Using a combination of liquid chromatography and electrospray ionization tandem mass spectrometry, we quantified 328 lipid species from 24 lipid classes and subclasses in 100 male adolescents aged 14–16 years who were categorized into four groups: (1) normal weight with traditional normal clinical plasma lipid levels (NN); (2) normal weight with traditional abnormal clinical plasma lipid levels (NA); (3) obese with traditional normal clinical plasma lipid levels (ON); and (4) obese with traditional abnormal clinical plasma lipid levels (OA). The concentrations of all the lipid species were compared between obese and normal-weight adolescents at different traditional clinical plasma lipid levels using the Kruskal–Wallis test followed by the Mann–Whitney *U* test. A partial least squares discriminant analysis (PLS-DA) was applied to select lipids with a significant ability to discriminate adolescent obesity.

**Results:**

The lipidomic profile distinguished obese adolescents from normal-weight subjects. Regardless of whether traditional clinical plasma lipid levels were normal or abnormal, we observed a significant reduction in the levels of five lysophosphatidylcholines (LPC) species (LPC18:2, LPC18:1, LPC20:2, LPC20:1, and LPC20:0) in the obese group compared with the normal-weight group (difference = −31.29% to −13.19%; *P*=9.91 × 10^−5^ to 2.28 × 10^−2^). The ability of these five LPC species to discriminate adolescent obesity was confirmed in the PLS-DA model.

**Conclusions:**

The findings provided evidence for the association of some LPC species with adolescent obesity. The discriminatory effects of five LPC species were identified between normal-weight and obese adolescents, independent of traditional clinical plasma lipid levels. These results will provide a basis for validation in subsequent studies.

## 1. Introduction

The prevalence of obesity in children and adolescents has been increasing worldwide [1, 2]. Over 340 million children and adolescents aged 5–19 are overweight or obese (World Health Organization, 2018). Obesity at younger ages adversely affects the metabolic health of young people and results in a variety of severe diseases in childhood and adulthood, including type 2 diabetes mellitus (T2DM), metabolic syndrome, atherosclerosis, and cardiovascular disease [3–5]. Although the precise mechanism linking obesity to these diseases remains unclear, extensive evidence suggests that dyslipidaemia plays an important role [6]. As a growing epidemic, obesity prompts the need to detect early metabolic changes using better biomarkers for early prevention.

Traditional clinical biochemistry uses measurements of cholesterol (TC), triglyceride (TG), low-density lipoprotein cholesterol (LDL-C), and high-density lipoprotein cholesterol (HDL-C) levels as tools to identify the health status and disease risk. Obesity and related diseases are closely associated with traditional dyslipidaemia, as defined by increasing TC, TG, and LDL-C levels or decreasing HDL-C levels. However, not all obese individuals present with dyslipidaemia and not all patients with dyslipidaemia are obese. After the application of lipidomics, researchers have observed the complexity of the plasma lipidome and performed in-depth analyses of the relationship between the lipid profile and obesity. Lipidomics is the systematic identification of the lipid species present in a cell, tissue, biofluid, or whole organism, which is known as a frontier of omics research and provides possibilities to identify next-generation biomarkers for complex diseases [7, 8]. The lipidome represents the intermediate product and end product of lipid metabolism that reflects physiological dysfunction and mirrors earlier stages of changes in metabolism. Therefore, lipidomic studies might be able to detect the obesity-related metabolic perturbation at an early stage, which would help to identify new biomarkers.

The major families of plasma lipids include fatty acids, sterol lipids, glycerolipids, glycerophospholipids, sphingolipids, and prenol lipids [9]. In recent years, a few studies have examined these plasma lipids associated with obesity or metabolic disorders [10–26]. According to Rodriguez-Cuenca et al., the dysregulation of the balance between sphingolipids and glycerophospholipids results in a lipotoxic insult relevant to the pathophysiology of common metabolic diseases, such as obesity, diabetes, or nonalcoholic fatty liver disease (NAFLD) [10]. An intervention study found that hydroxypropionic (hydroxyphenyllactic-related) acids, amino acids, and glycolipids were the most significant clusters of metabolites altered after bariatric surgery in morbidly obese subjects [11]. Although it has been reported that lysophosphatidylcholines (LPC) was associated with obesity, the results were not consistent in different studies [12–26]. As shown in the study by Wahl et al., the levels of LPC18:1, LPC18:2, and LPC20:4 are significantly decreased in obese children compared with nonobese children [12]. The trend was not influenced by weight loss in obese groups [13]. Additionally, significantly lower concentrations of lysophospholipids were observed in obese children than in nonobese children [14]. Some studies of adult subjects have suggested that certain LPC species, particularly unsaturated LPC species, were negatively correlated with BMI, obesity, or waist circumference [15–23]. In addition, negative correlations of some LPC species were not only observed with obesity but also with C-reactive protein, implying that some LPC species potentially exert anti-inflammatory effects [17, 23]. However, a monozygotic twin study revealed a correlation between BMI and increased levels of LPC16:0 and LPC18:0, regardless of genetic factors [24]. Similarly, Bagheri et al. identified a higher level of LPC16:1 in the obese group, while other LPC species were present at higher levels in nonobese subjects [26]. Thus, the associations between obesity and LPC remain controversial and therefore must be further validated in different populations. To the best of our knowledge, no study has investigated the associations of the plasma lipidome with adolescent obesity in subjects with normal or abnormal traditional clinical plasma lipid levels.

We performed the present study to investigate the associations of the plasma lipidome with obesity in Chinese adolescents and compare the lipidomic profiles between obese and normal-weight adolescents independent of traditional dyslipidaemia indexes, which will provide some clues for the discovery of new biomarkers in future validation studies.

## 2. Materials and Methods

### 2.1. Study Population and Anthropometric Measurements

From April to June 2012, we selected 50 obese boys aged 14–16 years (one half with abnormal clinical lipid levels and the other half with normal clinical lipid levels) and 50 normal-weight boys with a similar age distribution and plasma lipid levels. The 100 boys of Han nationality were recruited from 1000 students in Dongcheng District, Beijing, when they participated in the usual health examination. Adolescents with metabolic disorders such as T2DM and cardiovascular diseases or who had received drug therapy for hyperlipidaemia were excluded.

All selected adolescents were categorized into four study groups: (1) normal weight with traditional normal clinical plasma lipid levels (NN); (2) normal weight with traditional abnormal clinical plasma lipid levels (NA); (3) obese with traditional normal clinical plasma lipid levels (ON); and (4) obese with traditional abnormal clinical plasma lipid levels (OA). The study was approved by the ethics committee of Peking University Health Science Centre. Written informed consent was provided by all participants and their parents.

Anthropometric measurements, including height and weight, were measured by trained health professionals using standard protocols. We used the BMI percentile criteria to define obese and normal-weight adolescents, which were determined in a representative Chinese population [27]. The criteria are described in the appendix file [Supplementary-material supplementary-material-1]. The male adolescents with an age- specific BMI ≥ the 95th percentile were defined as obese and adolescents with a BMI between 15th and 85th percentiles were considered normal weight.

### 2.2. Metabolic Measurements

Venous blood samples were collected in the morning after an overnight fast. TC, TG, LDL-C, and HDL-C levels were measured in the blood samples with a biochemical auto-analyser (Hitachi 7060, Tokyo, Japan). Traditional abnormal clinical plasma lipid levels were defined in this study as abnormal levels of at least 2 of the 4 lipids: TG ≥1.10 mmol/L; TC ≥4.403 mmol/L; LDL-C ≥2.85 mmol/L; and HDL-C ≤1.17 mmol/L. These cutoff values were chosen based on the cholesterol levels in children and adolescents developed by the expert panel of the National Cholesterol Education Program (NCEP) [28].

Then, the lipidomic profiles of plasma samples were detected at Baker IDI Heart and Diabetes Institute, Australia. We quantified 328 lipid species from 24 lipid classes and subclasses (descriptive statistics are shown in the Additional file [Supplementary-material supplementary-material-1]) in 10 *μ*L of plasma using liquid chromatography coupled with electrospray ionization tandem mass spectrometry (LC ESI-MS/MS). The methods have been described in detail in a previous study [29]. Briefly, 10 *μ*L of plasma was mixed with 200 *μ*L of CHCl_3_/MeOH (2 : 1) and 10 *μ*L of internal standards (stable isotope-labelled and nonphysiological lipid species). These samples were mixed (rotary mixer, 10 min), sonicated (water bath, 30 min), and incubated (20 min) at room temperature. Then, the samples were centrifuged (16,000 × *g*, 10 min), and the supernatant was dried under a stream of nitrogen at 40°C. Extracted lipids were resuspended in 50 *μ*L of H_2_O-saturated BuOH, followed by 50 *μ*L of 10 mM NH_4_CHOO in MeOH. Extracts were centrifuged (3,350 × *g*, 5 min), and the supernatant was transferred to 0.2 mL glass inserts in vials with Teflon-lined caps.

The lipidomic analysis was performed using an Agilent 1200 HPLC system (Agilent Technologies, Santa Clara, CA, USA) coupled with an AB SCIEX API 4000 Q/TRAP mass spectrometer (AB Sciex, Framingham, MA, USA) with a turbo-ionspray source (350°C) and Analyst 1.5 and Multiquant data analysis systems. Internal standards were purchased from Sigma (St. Louis, MO, USA) and Matreya (Pleasant Gap, PA, USA) [30]. The levels of individual lipid species were measured using scheduled multiple-reaction monitoring in the positive ion mode [15, 31]. Lipid levels were calculated by relating the peak area of each species to the peak area of the corresponding internal standard. Peak integration was performed using AB Sciex MultiQuant software v1.2 [15].

### 2.3. Statistical Analyses

All analyses were performed with SPSS 18.0 and SIMCA 14.1 software. Continuous variables were summarized as means and standard deviations. Anthropometric characteristics of obese and normal-weight adolescents were compared with the Mann–Whitney *U* test. The concentrations of the 328 lipid species from 24 lipid classes and subclasses were compared between obese and normal-weight adolescents with different traditional clinical plasma lipid levels using the Kruskal–Wallis test followed by the Mann–Whitney *U* test. The associations of the plasma lipid profiles with adolescent obesity were investigated using a partial least squares discriminant analysis (PLS-DA). Data were visualized by plotting the scores of the first two components in a score plot where each point represents an individual plasma sample. Briefly, the variable importance in the projection (VIP) of the PLS-DA is a weighted sum of squares of the PLS loadings that considers the amount of *Y*-variation in each dimension. We calculated VIP values to select potential obesity-associated lipidome. Pearson correlation coefficients were calculated to determine the relationship between the levels of these lipids and traditional clinical parameters or anthropometric parameters (BMI).

## 3. Results

### 3.1. Characteristics of the Study Population

The demographic and traditional clinical plasma lipid levels of the adolescents are summarized in Table 1. No significant difference was observed in age among the groups. Obese adolescents had a significantly higher body weight and BMI than normal-weight adolescents (*P* < 0.001). The levels of TG, TC, LDL-C, and HDL-C differed significantly in the groups with abnormal plasma lipid levels (*P* < 0.001).

### 3.2. Discovery of the Lipids That Discriminate Adolescent Obesity

The levels of 328 lipid species from 24 classes and subclasses were measured in the 50 obese and the 50 normal-weight children. PC (1029.85 ± 105.98 *μ*mol/L), CE (979.72 ± 110.87 *μ*mol/L), COH (485.83 ± 76.38 *μ*mol/L), TG (157.56 ± 60.78 *μ*mol/L), SM (207.74 ± 22.08 *μ*mol/L), and LPC (155.74 ± 19.72 *μ*mol/L) were the most abundant lipids in the normal-weight normal plasma lipid level group. All other lipid classes were detected at levels lower than 150 *μ*mol/L.

**Figure 1:**
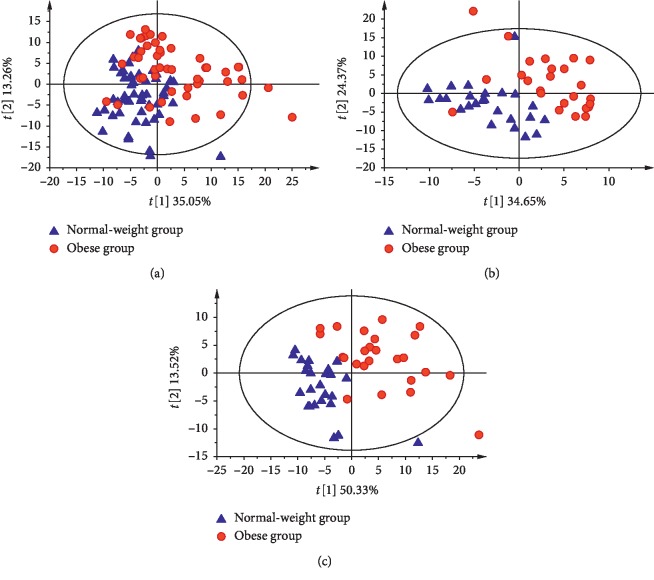
PLS-DA score plots based on the plasma levels of 328 lipid species in both the normal-weight group (blue) and obese group (red). Percentages in parentheses represent the variance in the lipidomic profile data obtained from the first two components. (a) PLS-DA: total (50 vs. 50). (b) PLS-DA: normal clinical lipid level (25 vs. 25). (c) PLS-DA: abnormal clinical lipid level (25 vs. 25).

The associations of the plasma lipid profiles with obesity were investigated by performing PLS-DA analyses of the 328 lipid species. The PLS-DA score plots are shown in Figures 1(a)–1(c). We observed a distinct separation of obese and normal-weight adolescents based on the first two components, which accounted for 35.05%, 13.26% (obese group vs. normal-weight group), 34.65%, 24.37% (ON vs. NN), 50.33%, and 13.52% (OA vs. NA) of the total variance in the lipidomic data, respectively.

The *R*^2^ and *Q*^2^ values of the PLS-DA models are presented in additional file [Supplementary-material supplementary-material-1]. All VIP parameters (VIP > 1) of PLS-DA models are presented in Table 2.

### 3.3. Difference in Plasma Lipid Profiles between the Obese and Normal-Weight Groups

**Figure 2:**
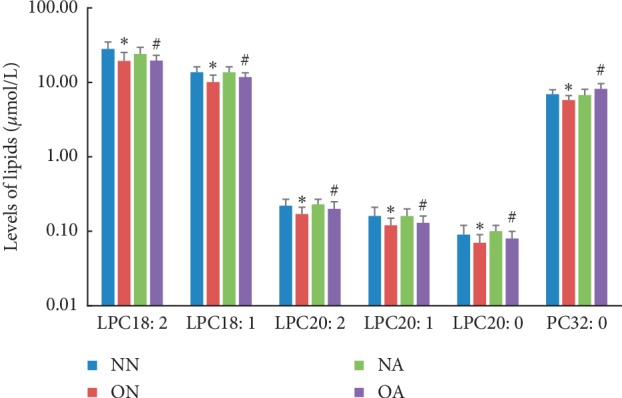
Lipid concentrations differed significantly between normal-weight and obese adolescents with different clinical lipid levels. ^*∗*^ON vs. NN, *P* < 0.05; ^#^OA vs. NA, *P* < 0.05. Abbreviations: NN, normal weight with traditional normal clinical plasma lipid levels; NA, normal weight with traditional abnormal clinical plasma lipid levels; ON, obese with traditional normal clinical plasma lipid levels; OA, obese with traditional abnormal clinical plasma lipid levels; LPC/PC *x: y*, (lyso) phosphatidylcholines with acyl chain length *x* and *y* double bonds.

The lipidomes were significantly different between obese groups and normal-weight groups of adolescents presenting both traditional normal and abnormal clinical plasma lipid levels, as shown in Figure 2 and the appendix file [Supplementary-material supplementary-material-1]. In the groups with traditional normal clinical plasma lipid levels, significantly lower levels of five lysophosphatidylcholines (LPC18:1, LPC18:2, LPC20:2, LPC20:1, and LPC20:0) were observed in the obese group than in the normal-weight group (difference = −16.32% to −31.29%; *P*=9.91 × 10^−5^ to 6.67 × 10^−3^). A similar pattern was also observed in the groups with traditional abnormal clinical plasma lipid levels. Markedly lower levels of five LPC species were observed in obese individuals than in normal-weight individuals (difference = −22.27% to −13.19%; *P*=4.40 × 10^−3^ to 2.28 × 10^−2^). Inverse correlations between LPC18:1, LPC18:2, LPC20:2, LPC20:1, and LPC20:0 and obesity were identified in groups with different traditional clinical plasma lipid levels. The observed reduction in the levels of the five LPC species in obese children was still significant after normalization to the total concentration of glycerophospholipids (i.e., PC, LPC, PE, PI, PG, PS, LPE, LPI, PC (O), PC (P), PE (O), and PE (P)). The results in detail are shown in the appendix file [Supplementary-material supplementary-material-1]. Additionally, in the obese group, an elevated phosphatidylcholine 32:0 (PC32:0) level (difference = 20.87%; *P*=4.40 × 10^−3^) was observed in adolescents with traditional abnormal clinical plasma lipid levels and decreased PC32:0 levels (difference = −16.32%; *P*=4.91 × 10^−4^) were observed in adolescents with traditional normal clinical plasma lipid levels in our study. The concentrations of other components of the lipidome and *P* values obtained from the Kruskal–Wallis test followed by the Mann–Whitney *U* test are summarized in the appendix file [Supplementary-material supplementary-material-1].

### 3.4. Identification of the Lipids That Discriminate Adolescent Obesity

The VIP parameter (VIP > 1) and Mann–Whitney *U* test (*P* < 0.05) were used to identify lipid species that significantly discriminated adolescent obesity. Five lipid species (LPC18:2, LPC18:1, LPC20:2, LPC20:1, and LPC20:0) met this criterion, as shown in Table 3. Interestingly, all 5 lipids were LPCs. The discriminatory ability of the lipids was not modulated by traditional clinical plasma lipid levels.

### 3.5. Associations of the Lipids That Discriminate Adolescent Obesity with Traditional Plasma Clinical Lipid Parameters

The results of correlation analyses between these discriminatory LPC lipids and traditional clinical parameters or anthropometric parameter (BMI) are shown in Table 4. Negative correlations of these discriminatory LPC lipids with BMI were observed. The levels of LPC18:2, LPC18:1, and LPC20:1 were positively correlated with the HDL-C levels. LPC18:2 level was positively correlated with TG level. LPC18:1 level was negatively correlated with TC level. Generally, obese people present elevated levels of TC, TG, and LDL-C and decreased levels of HDL-C. However, the correlations between the levels of the five LPC lipids and traditional plasma lipid indicators were not directionally consistent. Based on these results, the associations between the levels of the five LPC species and obesity were not explained by the association between traditional plasma lipid levels and obesity.

## 4. Discussion

A perturbation in lipid metabolism plays an important role in obesity-associated diseases. However, not all obese individuals have traditional abnormal clinical plasma lipid levels. Given the contradictory relationship, new lipid biomarkers for obesity must be discovered.

In the present study, we observed significantly decreased concentrations of unsaturated LPC, LPC18:2, LPC18:1, LPC20:2, LPC20:1, and LPC20:0 in obese adolescents, which were not affected by traditional clinical plasma lipid levels. A possible discriminatory effect of the five LPC species was identified between normal-weight and obese adolescents and was independent of traditional plasma lipid indicators. These findings will contribute to the identification of new biomarkers in future studies.

Similar results have been reported in other studies of the lipidomic profiles of obese children [12, 13]. Significantly decreased levels of LPC18:1 and LPC18:2 have been observed in obese children [12]. The same trend was also observed in obese adolescents who lost weight after participating in a lifestyle intervention [13]. Although the lipid data from adults and children are difficult to compare, consistent changes in the levels of some LPC species related to obesity have been identified [15–23]. A large population-based cohort study showed a strong negative correlation between the total LPC levels and BMI, although the authors did not further explore the LPC species [15]. Rauschert et al. reported negative correlations between LPC18:1 and LPC18:2 with waist circumference that were independent of the LDL-C and HDL-C levels [16]. Wang et al. also observed lower concentrations of unsaturated LPC, LPC18:1, and LPC18:2 in overweight/obese adults than in nonobese adults [17]. Heimerl et al. detected significant negative correlations between BMI and the levels of unsaturated LPC, LPC15:0, LPC18:1, LPC18:2, LPC18:3, LPC20:0, and LPC22:6 [18]. Tulipani et al. concluded that plasma concentrations of LPC17:0, LPC18:1, and LPC18:2 show inverse correlations with BMI, body weight, and waist and hip circumference in morbidly obese subjects [19, 20]. Notably, decreased plasma concentrations of LPC18:1 and LPC18:2 have been detected in both subjects with metabolically unhealthy obesity and metabolically healthy obesity [21]. In addition, lower total LPC, LPC18:0, LPC18:2, and LPC20:4 levels were measured in obese and obese subjects with type 2 diabetes than in nonobese adults. A difference in the LPC profile was not observed between obese individuals and obese subjects with type 2 diabetes [22]. Moreover, Wallace et al. reported associations between the levels of several LPC species, BMI, and inflammatory markers [23]. Additionally, LPC levels were significantly negatively correlated with the levels of C-reactive protein (CRP), indicating that LPC may exert anti-inflammatory effects [18, 23]. The levels of some LPC species appear to be altered, although the direction of changes is not consistent. A monozygotic twin study observed correlations between BMI and increased levels of LPC16:0 and LPC18:0 that were independent of genetic factors [24]. The authors identified higher levels of LPC14:0 and LPC18:0 and a lower concentration of LPC18:1 in obese subjects compared with lean subjects [25]. A recent study by Bagheri et al. identified a metabolic pattern associated with obesity. Based on this pattern, a higher LPC 16:1 level was detected in the obese group, while higher levels of other LPC species were observed in nonobese subjects [26].

The present study extended the results from previous studies by revealing significant reductions in the levels of the unsaturated LPC subclass and five LPC species (LPC18:2, LPC18:1, LPC20:2, LPC20:1, and LPC20:0) in obese adolescents that were independent of changes in traditional clinical plasma lipid levels. Four of the five species are unsaturated. The mechanism underlying the reduction remains unknown but may be attributed to the decreased activity of lecithin cholesterol acyltransferase (LCAT). LPCs in human plasma are mainly derived from the activity of LCAT. LCAT removes fatty acids from the sn-1 or -2 position of PC to form LPC [32]. Thus, decreased activity of LCAT in obese subjects potentially leads to a decrease in circulating LPC levels [33, 34]. However, we have not measured LCAT activity in this study. It could be measured in further studies to explore this hypothesis.

The proinflammatory and anti-inflammatory effects of LPC are contradictory and poorly understood but may be due to acyl chain-dependent differences in biological activity. Unsaturated LPCs might be hydrolysed in vivo to release polyunsaturated fatty acids, which are hypothesized to possess anti-inflammation properties and exert positive effects on health, as confirmed by Hung et al. [35]. Based on these findings, unsaturated LPCs suppress the pro-inflammatory effect of saturated LPCs. The balance of unsaturated LPCs and saturated LPCs may play a role in the inflammatory response observed in obese individuals. In our study, most species of LPCs related to obesity that showed a decrease in abundance were unsaturated LPCs. In other words, the imbalance of unsaturated LPCs and saturated LPCs was also observed in our study. Overall, the relationship between the imbalance of LPCs and chronic inflammation, which plays an important role in obesity and obesity-related metabolic diseases, awaits further confirmation in studies employing larger sample sizes.

According to previous studies, most of the circulating LPCs are bound to albumin and do not interact with lipoproteins [36, 37]. In this study, the association between the levels of five LPC species and obesity was not explained by the association between traditional plasma lipid levels and obesity. Therefore, these LPC species may be early and specific indicators of obesity, independent of the traditional plasma lipids levels.

LPCs have been identified as important signalling molecules and have been proposed to be involved in regulating cellular proliferation, tumour cell invasion, and inflammation [38–40]. Some studies also discovered that LPCs exert beneficial effects on glucose metabolism. LPCs stimulate glucose uptake in adipocytes, potentiate glucose-stimulated insulin secretion, and lower blood glucose levels in rat models of diabetes [41, 42]. Thus, the reduction in circulating levels of LPC associated with obesity may contribute to the imbalance in glucose metabolism in obese individuals. The lipids with beneficial metabolic effects have recently received prominence [43].

The value of this study lies in the adoption of a systematic lipidomic approach to investigate the association of plasma levels of LPC species with adolescent obesity. Furthermore, research on the lipidomic profiles on adolescents might approach the “true” association between lipid levels and obesity more closely than research conducted in adults, because adolescents usually do not suffer from other diseases that alter the plasma lipid profiles [44]. Finally, but most importantly, we conducted stratified analyses according to the clinical lipid levels and matched obese groups with normal-weight groups presenting different plasma lipid levels, which facilitated the detection of new lipidome biomarkers for obesity.

Our study has some limitations. First, the sample size was small, and thus, smaller effects were difficult to identify. However, we only recruited boys and selected a small age range to reduce the effects of confounding factors [17]. Second, the cross-sectional design failed to reveal the causal relationship between lipid levels and obesity. Furthermore, the study lacked information about the children's diet and physical activity. However, in previous cross-sectional lipidomics studies, physical activity and dietary habits were not adjusted as confounding factors [13, 18, 22, 23], suggesting that the abnormal lipid profile might be an independent risk factor for obesity. This hypothesis should be validated in further studies.

## 5. Conclusions

In conclusion, we identified the discriminatory effects of five LPC species (LPC18:2, LPC18:1, LPC20:2, LPC20:1, and LPC20:0) on obese and normal-weight adolescents that were independent of traditional clinical plasma lipid levels. Most of these discriminatory LPC species belonged to the unsaturated LPC subclass. The results will provide opportunities for future validation studies.

## Figures and Tables

**Table 1:** Demographic and clinical characteristics^a^ of the male adolescents.

	Normal-weight group (*n* = 50)	Obese group (*n* = 50)	NN group (*n* = 25)	NA group (*n* = 25)	ON group (*n* = 25)	OA group (*n* = 25)
Age (years)	14.68 ± 0.47	14.72 ± 0.61	14.68 ± 0.48	14.68 ± 0.48	14.72 ± 0.61	14.72 ± 0.61
Weight (kg)	59.31 ± 6.27	93.38 ± 12.53^*∗∗*^	58.39 ± 4.96	60.22 ± 7.35	87.71 ± 10.1	99.0 ± 12.31^&^
BMI (kg/m^2^)	19.85 ± 1.63	30.80 ± 3.93^*∗∗*^	19.56 ± 1.21	20.13 ± 1.94	29.50 ± 3.91	32.10 ± 3.56^&^
TG (mmol/L)	0.93 ± 0.50	1.27 ± 0.84	0.57 ± 0.20	1.29 ± 0.46^##^	0.62 ± 0.23	1.92 ± 0.71^&&^
TC (mmol/L)	3.77 ± 0.79	4.31 ± 1.32	3.22 ± 0.29	4.33 ± 0.73^##^	3.14 ± 0.42	5.48 ± 0.73^&&^
LDL-C (mmol/L)	3.13 ± 1.03	3.91 ± 1.75^*∗*^	2.30 ± 0.28	3.96 ± 0.81^##^	2.33 ± 0.53	5.50 ± 0.88^&&^
HDL-C (mmol/L)	1.40 ± 0.29	1.25 ± 0.27^*∗*^	1.56 ± 0.25	1.25 ± 0.23^##^	1.42 ± 0.21	1.09 ± 0.22^&&^

^a^All values are presented as the means ± SD. TG, triglyceride; TC, total cholesterol; LDL-C, low-density lipoprotein cholesterol; HDL-C, high-density lipoprotein cholesterol; NN, normal weight with traditional normal clinical plasma lipid levels; NA, normal weight with traditional abnormal clinical plasma lipid levels; ON, obese with traditional normal clinical plasma lipid levels; OA, obese with traditional abnormal clinical plasma lipid levels; BMI, body mass index. Traditional abnormal clinical plasma lipid levels were defined as abnormal levels of at least 2 of the 4 lipids: TG ≥ 1.1 mmol/L; TC ≥ 4.403 mmol/L; LDL-C ≥ 2.85 mmol/L; and HDL-C ≤ 1.17 mmol/L. ^*∗∗*^Obese group vs. normal-weight group *P* < 0.001; ^*∗*^obese group vs. normal-weight group *P* < 0.05; ^##^NA vs. NN *P* < 0.001; ^&&^OA vs. ON *P* < 0.001; ^&^OA vs. ON *P* < 0.05.

**Table 2:** Summary of all significant variable importance in the projection (VIP)^a^ values identified in the partial least squares discriminant analysis (PLS-DA).

	O (50) vs. N (50)	ON (*n* = 25) vs. NN (*n* = 25)	OA (*n* = 25) vs. NA (*n* = 25)
	VIP > 1	VIP > 1	VIP > 1
CE16:0	1.35	1.06	2.05
CE16:2	1.46	1.06	1.95
CE17:1	1.34	1.05	2.07
CE18:0	1.05	1.42	1.22
CE20:2	1.71	1.2	2.03
CE20:3	1.75	1.04	2.09
CE20:4	1.18	1.15	1.45
CE20:5	1.44	1.07	1.63
CE22:6	1.15	1.37	1.13
dhCer20:0	1.42	1.78	1.2
LPC18:1	2.33	2.22	1.59
LPC18:2	2.21	2.21	1.44
LPC20:0	1.94	1.79	1.32
LPC20:1	2.01	1.62	1.5
LPC20:2	1.91	1.8	1.18
LPC26:0	1.57	1.68	1.05
oddPC31:1	1.17	1.41	1.13
PC (18:2/20:4)	1.23	1.31	1.04
PC36:1	1.18	1.15	1.58
PC38:3	1.49	1.21	1.77
PC40:6	1.25	1.23	1.2
PC40:8	1.42	1.22	1.28
PE (16:0/0:0)	1.67	1.84	1.19
PE (18:0/0:0)	1.57	1.71	1.25
PE (20:4/0:0)	1.76	1.36	1.58
PE (O-34:2)	1.48	1.13	1.27

^a^A significant VIP was defined as VIP > 1. N, normal-weight group; O, obese group; NN, normal weight with traditional normal clinical plasma lipid levels; NA, normal weight with traditional abnormal clinical plasma lipid levels; ON, obese with traditional normal clinical plasma lipid levels; OA, obese with traditional abnormal clinical plasma lipid levels.

**Table 3:** Potential biomarkers of adolescent obesity identified in the lipidomic analysis of normal-weight groups and obese groups with different clinical plasma lipid levels (*P* < 0.05).

	O (50) vs. N (50)		ON (*n* = 25) vs. NN (*n* = 25)		OA (*n* = 25) vs. NA (*n* = 25)	
	VIP	*P* value	VIP	*P* value	VIP	*P* value
LPC18:2	2.21	6.74 × 10^−8*∗∗*^	2.21	1.08 × 10^−4*∗∗*^	1.44	2.00 × 10^−2*∗*^
LPC18:1	2.33	4.04 × 10^−7*∗∗*^	2.22	9.91 × 10^−5*∗∗*^	1.59	2.00 × 10^−2*∗*^
LPC20:2	1.91	1.92 × 10^−7*∗∗*^	1.80	4.40 × 10^−3*∗∗*^	1.18	3.00 × 10^−2*∗*^
LPC20:1	2.01	2.33 × 10^−6*∗∗*^	1.62	6.67 × 10^−3*∗∗*^	1.50	4.00 ×
LPC20:0	1.94	3.61 × 10^−6*∗∗*^	1.79	7.33 × 10^−4*∗∗*^	1.32	2.00 × 10^−2*∗*^

^*∗*^ *P* < 0.05 and ^*∗∗*^*P* < 0.001. NN, normal weight with traditional normal clinical plasma lipid levels; NA, normal weight with traditional abnormal clinical plasma lipid levels; ON, obese with traditional normal clinical plasma lipid levels; OA, obese with traditional abnormal clinical plasma lipid levels; LPC *x*: *y*, lysophosphatidylcholines with acyl chain length *x* and *y* double bonds.

**Table 4:** Correlations of lipids discriminating adolescent obesity with traditional clinical parameters.

	HDL-C		TC		TG		LDL-C		BMI	
	*r*	*P* value	*r*	*P* value	*r*	*P* value	*r*	*P* value	*r*	*P* value
LPC18:2	**0.34** ^*∗*^	**4.63 × 10** ^**−4**^	−0.08	4.10 × 10^−1^	−**0.22**^*∗*^	**3.02 × 10** ^**−2**^	−0.13	1.96 × 10^−1^	−**0.47**^*∗*^	**7.12 × 10** ^**−7**^
LPC18:1	**0.24** ^*∗*^	**1.72 × 10** ^**−2**^	**0.21** ^*∗*^	**3.24 × 10** ^**−2**^	0.09	3.71 × 10^−1^	0.15	1.46 × 10^−1^	−**0.43**^*∗*^	**1.03 × 10** ^**−5**^
LPC20:2	0.11	2.75 × 10^−1^	0.19	6.43 × 10^−2^	0.07	5.07 × 10^−1^	0.01	1.39 × 10^−1^	−**0.33**^*∗*^	**8.50 × 10** ^**−4**^
LPC20:1	**0.23** ^*∗*^	**2.13 × 10** ^**−2**^	0.05	6.10 × 10^−1^	−0.11	2.72 × 10^−1^	0.01	9.05 × 10^−1^	−**0.39**^*∗*^	**4.91 × 10** ^**−5**^
LPC20:0	0.15	1.43 × 10^−1^	0.13	2.02 × 10^−1^	−0.02	8.27 × 10^−1^	0.09	3.89 × 10^−1^	−**0.35**^*∗*^	**3.12 × 10** ^**−4**^

^*∗*^ *P* < 0.05. TG, triglyceride; TC, total cholesterol; LDL-C, low-density lipoprotein cholesterol; HDL-C, high-density lipoprotein cholesterol. The values in bold denote statistical significance.

## Data Availability

The data used to support the findings of this study are restricted by the ethics committee in order to protect privacy of subjects. The data are available from the corresponding author upon reasonable request.

## Abbreviations

LPC: Lysophosphatidylcholines
LPC *x*: *y*: Lysophosphatidylcholines with acyl chain length *x* and *y* double bonds
NN: Normal weight with traditional normal clinical plasma lipid levels
NA: Normal weight with traditional abnormal clinical plasma lipid levels
ON: Obese with traditional normal clinical plasma lipid levels
OA: Obese with traditional abnormal clinical plasma lipid levels
PLS-DA: A partial least squares discriminant analysis
TC: Cholesterol
TG: Triglycerides
LDL-C: Low-density lipoprotein cholesterol
HDL-C: High-density lipoprotein cholesterol
T2DM: Type 2 diabetes mellitus
VIP: The variable important in projection.

## Supplementary Materials

Supplementary Materials Supplementary Table S1: the body mass index reference for screening overweight and obesity in Chinese boys aged 13–18 years. Supplementary Table S2: the *R*^2^and *Q*^2^ values of these partial least square discriminant analysis (PLS-DA) models. Supplementary Table S3: the lipid levels significantly differed between normal-weight and obese adolescents in different clinical lipid levels. Supplementary Table S4: 328 lipids from 24 classes were described and compared in different groups.
