# Supplementary material for: Lipidomic Profile Revealed the Association of Plasma Lysophosphatidylcholines with Adolescent Obesity
Source: Biomed Res Int. 2019 Dec 13;2019:1382418. doi: 10.1155/2019/1382418 (PMC6930386; doi:10.1155/2019/1382418)
Supplement: Supplementary Materials — Supplementary Table S1: the body mass index reference for screening overweight and obesity in Chinese boys aged 13–18 years. Supplementary Table S2: the R2and Q2 values of these partial least square discriminant analysis (PLS-DA) models. Supplementary Table S3: the lipid levels significantly differed between normal-weight and obese adolescents in different clinical lipid levels. Supplementary Table S4: 328 lipids from 24 classes were described and compared in different groups. [file 1382418.f1.pdf]

Table S1: The body mass index reference for screening overweight and obesity in Chinese boys aged 13-18 years [20]

Age (years)	Overweight	Obesity
13-	21.9	25.7
14-	22.6	26.4
15-	23.1	26.9
16-	23.5	27.4
17-	23.8	27.8
18	24.0	28.0

Table S2: The R^2 and Q^2 values of these partial least squares discriminant analysis (PLS-DA) models

		R^2X(cum)	R^2Y(cum)	Q^2(cum)
O（50）vs. N (50)	1	0.14	0.35	0.24
	2	0.36	0.48	0.33
	3	0.48	0.59	0.40
ON（n=25）vs. NN（n=25）	1	0.19	0.35	0.11
	2	0.31	0.59	0.21
OA（n=25）vs. NA（n=25）	1	0.18	0.53	0.43
	2	0.32	0.69	0.51

a Abbreviations: N, Normal-weight; O, obese; NN, Normal-weight with traditional Normal clinical plasma lipid levels; NA, Normal- weight with traditional Abnormal clinical plasma lipid levels; ON, Obese with traditional Normal clinical plasma lipid levels; OA, Obese with traditional Abnormal clinical plasma lipid levels.

Table S3: The lipids levels significantly differed between normal-weight and obese adolescents in different clinical lipid levels [a]

	NN	NA	ON	OA	P^c	p^d	ON vs. NN			OA vs. NA		
							difference (%) [b]	*P*	p^d	difference (%) [b]	*P*	p^d
**LPC 18:2**	28.16±6.79	24.00±5.60	19.35±5.84	19.61±3.48	8.07×10^{-7}	4.07×10^{-10}	-31.29	1.08×10^{-4}	7.87×10^{-5}	-18.14	2.02×10^{-2}	7.74×10^{-6}
**LPC 18:1**	13.66±2.53	13.69±2.51	10.11±2.4	11.76±1.69	2.39×10^{-6}	1.09×10^{-9}	-26.01	9.91×10^{-5}	4.42×10^{-5}	-14.13	2.02×10^{-2}	2.54×10^{-6}
**LPC 20:2**	0.22±0.05	0.23±0.04	0.17±0.04	0.20±0.05	7.58×10^{-5}	1.12×10^{-5}	-23.15	4.40×10^{-3}	2.72×10^{-3}	-14.69	3.47×10^{-2}	1.38×10^{-4}
**LPC 20:1**	0.16±0.05	0.16±0.04	0.12±0.03	0.13±0.03	7.28×10^{-5}	2.12×10^{-6}	-22.88	6.67×10^{-3}	1.57×10^{-2}	-22.27	4.40×10^{-3}	3.16×10^{-5}
**LPC 20:0**	0.09±0.03	0.10±0.02	0.07±0.02	0.08±0.02	1.33×10^{-5}	4.14×10^{-6}	-27.66	7.33×10^{-4}	4.21×10^{-3}	-18.36	2.28×10^{-2}	6.16×10^{-5}
**PC 32:0**	6.93±1.03	6.75±1.35	5.80±0.85	8.16±1.49	2.25×10^{-7}	4.51×10^{-3}	-16.32	4.91×10^{-4}	9.06×10^{-3}	20.87	4.40×10^{-3}	3.05×10^{-2}

a All values are presented as the means±SD of plasma lipid concentration in umol/L.

b Percentage (%) of difference between obese and normal-weight adolescents.

c We only listed lipidome whose concentrations differed significantly after Benjamini-Hochberg correction for multiple testing.

d The P-value was presented after the normalization with the total concentration of glycerophospholipids.

e Abbreviations: NN, Normal-weight with traditional Normal clinical plasma lipid levels; NA, Normal-weight with traditional Abnormal clinical plasma lipid levels; ON, Obese with traditional Normal clinical plasma lipid levels; OA, Obese with traditional Abnormal clinical plasma lipid levels; LPC x: y, lysophosphatidylcholines with acyl chain length x and y double bonds.

Table S4: 328 lipids from 24 classes were described and compared in different groups [a]

	NN	NA	ON	OA	P^{c}	ON vs. NN difference (%) [b]	P	OA vs. NA difference (%) [b]	P
Total dhCer	0.39±0.07	0.42±0.12	0.54±0.11	0.82±0.26	**1.37×10^{-11}**	6.80	2.20×10^{-1}	52.42	**1.53×10^{-4}**
dhCer 16:0	0.05±0.01	0.04±0.01	0.04±0.01	0.05±0.01	**1.13×10^{-11}**	-9.46	6.40×10^{-1}	19.52	**4.62×10^{-2}**
dhCer 20:0	0.02±0.01	0.02±0.01	0.02±0.01	0.03±0.01	**2.19×10^{-6}**	26.08	1.74×10^{-1}	32.46	**4.37×10^{-2}**
dhCer 22:0	0.08±0.02	0.09±0.03	0.12±0.03	0.19±0.07	**9.76×10^{-11}**	10.96	3.33×10^{-1}	60.97	**2.77×10^{-4}**
dhCer 24:1	0.07±0.02	0.07±0.03	0.10±0.02	0.14±0.05	**8.91×10^{-10}**	8.52	9.50×10^{-1}	51.37	**4.91×10^{-4}**
dhCer 24:0	0.18±0.04	0.20±0.06	0.26±0.06	0.41±0.15	**6.37×10^{-11}**	6.77	8.75×10^{-1}	56.09	**2.54×10^{-4}**
Total Cer	6.43±1.35	5.67±1.45	8.80±1.63	9.61±1.58	**2.22×10^{-11}**	-11.80	7.00×10^{-2}	9.23	6.00×10^{-2}
Cer 16:0	0.23±0.04	0.20±0.05	0.28±0.05	0.30±0.06	**1.06×10^{-7}**	-14.22	**3.90×10^{-2}**	7.95	8.47×10^{-1}
Cer 18:0	0.09±0.04	0.09±0.03	0.11±0.03	0.14±0.05	**5.27×10^{-6}**	-1.48	1.00×10^{0}	33.45	7.96×10^{-2}
Cer 20:0	0.10±0.03	0.09±0.03	0.12±0.03	0.14±0.04	**2.10×10^{-7}**	-9.65	6.97×10^{-1}	15.15	6.26×10^{-1}
Cer 22:0	0.93±0.22	0.86±0.24	1.27±0.30	1.49±0.38	**1.07×10^{-9}**	-7.57	8.02×10^{-1}	17.35	1.66×10^{-1}
Cer 24:1	1.04±0.29	0.89±0.24	1.40±0.30	1.43±0.35	**1.64×10^{-8}**	-14.49	1.98×10^{-1}	1.89	1.00×10^{0}
Cer 24:0	4.04±0.92	3.55±1.00	5.61±1.17	6.10±1.03	**8.21×10^{-11}**	-12.23	4.80×10^{-1}	8.70	4.37×10^{-1}
Total MHC	7.35±1.72	7.23±1.59	9.60±2.69	10.07±3.23	**7.75×10^{-5}**	-1.64	8.70×10^{-1}	4.94	9.60×10^{-1}
MHC 16:0	0.73±0.14	0.72±0.17	0.94±0.24	1.02±0.31	**4.73×10^{-6}**	-1.02	1.00×10^{0}	8.75	9.97×10^{-1}
MHC 18:0	0.11±0.03	0.10±0.02	0.13±0.04	0.13±0.03	**8.46×10^{-3}**	-2.92	1.00×10^{0}	-1.04	1.00×10^{0}
MHC 20:0	0.12±0.04	0.11±0.03	0.16±0.05	0.16±0.06	**6.96×10^{-4}**	-4.77	1.00×10^{0}	-0.02	1.00×10^{0}
MHC 22:0	1.78±0.45	1.79±0.42	2.38±0.77	2.50±0.76	**1.02×10^{-4}**	0.94	9.93×10^{-1}	5.05	9.97×10^{-1}

MHC 24:1	1.67±0.51	1.65±0.50	2.13±0.62	2.21±0.98	**5.26×10^{-3}**	-1.19	1.00×10^{0}	3.85	9.93×10^{-1}
MHC 24:0	2.95±0.73	2.85±0.59	3.86±1.17	4.05±1.28	**4.66×10^{-5}**	-3.42	1.00×10^{0}	4.97	9.99×10^{-1}
Total DHC	4.18±0.89	4.28±0.89	4.91±1.19	5.17±1.48	**1.18×10^{-2}**	2.28	6.20×10^{-1}	5.25	7.50×10^{-1}
DHC 16:0	3.32±0.73	3.44±0.71	3.96±0.97	4.18±1.22	**7.82×10^{-3}**	3.48	9.72×10^{-1}	5.46	9.99×10^{-1}
DHC 18:0	0.05±0.02	0.04±0.01	0.05±0.02	0.06±0.02	**4.10×10^{-2}**	-9.63	7.11×10^{-1}	22.38	2.98×10^{-1}
DHC 20:0	0.03±0.01	0.03±0.01	0.03±0.01	0.03±0.01	6.61×10^{-1}	3.85	1.00×10^{0}	2.43	9.93×10^{-1}
DHC 22:0	0.12±0.03	0.12±0.03	0.14±0.05	0.15±0.03	**2.12×10^{-2}**	-2.36	1.00×10^{0}	7.16	7.25×10^{-1}
DHC 24:1	0.52±0.15	0.51±0.15	0.57±0.17	0.58±0.23	5.87×10^{-1}	-0.74	9.99×10^{-1}	2.44	1.00×10^{0}
DHC 24:0	0.14±0.04	0.13±0.03	0.17±0.05	0.17±0.04	**3.62×10^{-3}**	-6.96	9.64×10^{-1}	3.98	9.96×10^{-1}
Total THC	1.52±0.25	1.33±0.30	1.52±0.36	1.49±0.38	9.82×10^{-2}	-12.38	1.00×10^{-1}	-2.13	8.50×10^{-1}
THC 16:0	0.76±0.12	0.66±0.14	0.77±0.18	0.77±0.17	2.98×10^{-2}	-12.39	**4.89×10^{-2}**	-0.99	1.00×10^{0}
THC 20:0	0.04±0.02	0.04±0.01	0.04±0.02	0.04±0.02	9.12×10^{-1}	-5.17	1.00×10^{0}	-8.87	9.81×10^{-1}
THC 22:0	0.21±0.05	0.19±0.06	0.20±0.06	0.19±0.06	5.70×10^{-1}	-8.81	8.36×10^{-1}	-6.84	9.68×10^{-1}
THC 24:1	0.30±0.07	0.27±0.08	0.30±0.09	0.29±0.12	2.99×10^{-1}	-10.73	3.21×10^{-1}	-3.03	9.79×10^{-1}
THC 24:0	0.21±0.04	0.17±0.04	0.20±0.06	0.20±0.06	**3.09×10^{-2}**	-19.58	8.74×10^{-3}	1.06	1.00×10^{0}
Total GM3	2.32±0.32	2.20±0.37	2.73±0.46	3.01±0.62	**2.84×10^{-7}**	-5.18	2.30×10^{-1}	9.90	1.70×10^{-1}
GM3 16:0	0.76±0.11	0.71±0.13	0.83±0.15	0.89±0.18	**2.23×10^{-4}**	-6.68	2.45×10^{-1}	7.77	9.19×10^{-1}
GM3 18:0	0.30±0.07	0.29±0.07	0.34±0.09	0.38±0.1	**3.31×10^{-3}**	-4.07	9.86×10^{-1}	12.74	8.14×10^{-1}
GM3 20:0	0.12±0.03	0.12±0.02	0.14±0.03	0.17±0.04	**3.77×10^{-6}**	1.67	9.99×10^{-1}	17.29	2.07×10^{-1}
GM3 22:0	0.34±0.06	0.34±0.08	0.44±0.1	0.55±0.13	**1.14×10^{-9}**	-0.11	1.00×10^{0}	23.53	**2.74×10^{-2}**
GM3 24:1	0.46±0.11	0.42±0.09	0.57±0.13	0.57±0.18	**6.98×10^{-5}**	-8.34	7.90×10^{-1}	1.62	1.00×10^{0}
GM3 24:0	0.35±0.08	0.33±0.08	0.41±0.08	0.44±0.11	**5.61×10^{-5}**	-5.94	8.91×10^{-1}	5.93	9.50×10^{-1}

Total SM	207.74±22.08	206.03±35.34	241.86±38.07	296.86±53.32	**3.85×10^{-10}**	-0.82	3.50×10^{-1}	22.74	**6.35×10^{-7}**
SM 31:1	0.08±0.02	0.09±0.03	0.10±0.03	0.13±0.04	**2.93×10^{-5}**	6.21	9.50×10^{-1}	21.32	3.09×10^{-1}
SM 32:2	0.40±0.08	0.46±0.1	0.47±1.18	0.69±0.17	**1.07×10^{-9}**	14.20	1.08×10^{-1}	46.74	**4.08×10^{-6}**
SM 32:1	3.79±0.93	4.06±1.16	4.73±1.18	6.27±1.85	**2.10×10^{-7}**	7.33	9.60×10^{-1}	32.45	**6.67×10^{-3}**
SM 32:0	0.08±0.03	0.09±0.03	0.11±0.03	0.16±0.08	**3.37×10^{-9}**	6.01	9.81×10^{-1}	41.85	**2.02×10^{-2}**
SM 33:1	2.56±0.53	2.63±0.69	3.11±0.81	3.60±0.82	**1.78×10^{-5}**	2.56	1.00×10^{0}	16.02	2.87×10^{-1}
SM 34:3	0.05±0.01	0.05±0.01	0.05±0.02	0.07±0.02	**2.17×10^{-5}**	11.20	7.11×10^{-1}	29.42	**2.67×10^{-3}**
SM 34:2	11.19±1,59	11.09±1.78	12.21±1.73	14.77±2.45	**1.89×10^{-7}**	-0.87	9.72×10^{-1}	20.98	**1.71×10^{-3}**
SM 34:1	75.34±9.93	71.23±13.92	84.29±15.79	94.9±17.97	**3.75×10^{-6}**	-5.46	3.71×10^{-1}	12.58	5.23×10^{-1}
SM 34:0	35.01±7.49	30.92±6.82	41.6±9.75	59.35±22.03	**8.81×10^{-9}**	-11.69	3.33×10^{-1}	42.67	**5.43×10^{-3}**
SM 35:2	0.28±0.06	0.29±0.07	0.32±0.06	0.37±0.07	**3.21×10^{-5}**	3.65	9.98×10^{-1}	18.41	**2.15×10^{-2}**
SM 35:1	1.34±0.28	1.44±0.36	1.68±0.42	1.88±0.38	**1.22×10^{-5}**	7.31	8.57×10^{-1}	11.57	4.51×10^{-1}
SM 36:3	0.52±0.10	0.54±0.12	0.57±0.1	0.68±0.13	**1.58×10^{-4}**	4.85	9.90×10^{-1}	17.53	**2.91×10^{-2}**
SM 36:2	6.44±1.48	7.29±1.58	7.95±1.58	9.76±2.28	**6.59×10^{-7}**	13.24	2.65×10^{-1}	22.79	**1.90×10^{-2}**
SM 36:1	10.62±2.11	12.41±3.05	14.06±3.01	17.61±3.64	**1.90×10^{-9}**	16.79	1.08×10^{-1}	25.22	**3.56×10^{-3}**
SM 37:2	0.11±0.03	0.13±0.05	0.14±0.04	0.16±0.05	**5.59×10^{-3}**	16.25	9.39×10^{-1}	14.62	5.38×10^{-1}
SM 38:2	4.4±0.88	4.35±0.72	4.78±0.69	5.17±0.95	**3.26×10^{-3}**	-1.15	1.00×10^{0}	8.20	7.77×10^{-1}
SM 38:1	11.09±2.12	11.68±2.93	12.63±2.32	14.25±2.85	**8.96×10^{-4}**	5.24	9.81×10^{-1}	12.73	3.21×10^{-1}
SM 39:1	3.32±0.68	3.53±0.78	3.58±0.8	4.07±0.78	**7.35×10^{-3}**	6.30	8.02×10^{-1}	13.71	1.81×10^{-1}
SM 41:2	8.94±1.30	8.92±1.53	9.50±1.33	10.42±1.99	**3.81×10^{-3}**	-0.27	1.00×10^{0}	9.73	5.96×10^{-1}
SM 41:1	10.40±1.64	11.08±2.44	12.64±2.22	16.72±3.19	**2.35×10^{-10}**	6.52	9.50×10^{-1}	32.22	**2.33×10^{-5}**
SM 42:1	21.77±3.33	23.77±4.81	27.3±5.53	35.82±7.24	**2.32×10^{-10}**	9.17	5.23×10^{-1}	31.21	**2.15×10^{-4}**

Total PC	1029.85±105.98	975.82±118.89	1152.73±140.1	1308.93±153.13	**1.31×10^{-9}**	-5.25	9.00×10^{-2}	13.55	**5.13×10^{-7}**
PC 28:0	0.05±0.04	0.04±0.02	0.06±0.04	0.13±0.12	**4.63×10^{-5}**	-28.97	9.68×10^{-1}	108.66	8.83×10^{-2}
PC 29:0	0.02±0.01	0.02±0.01	0.01±0.01	0.03±0.02	**2.98×10^{-2}**	-13.90	9.91×10^{-1}	42.40	6.11×10^{-1}
PC 30:0	0.86±0.39	0.75±0.24	1.08±0.37	1.71±0.86	**1.60×10^{-6}**	-13.30	9.94×10^{-1}	58.70	**1.57×10^{-2}**
PC 31:1	0.37±0.09	0.45±0.11	0.39±0.10	0.53±0.11	**2.53×10^{-6}**	7.76	5.96×10^{-1}	17.76	5.17×10^{-2}
PC 31:0	0.23±0.08	0.25±0.09	0.21±0.08	0.32±0.14	**1.31×10^{-2}**	-8.71	9.64×10^{-1}	25.19	3.97×10^{-1}
PC 32:3	0.17±0.04	0.17±0.04	0.18±0.03	0.24±0.05	**9.13×10^{-8}**	0.06	1.00×10^{0}	33.89	**8.31×10^{-5}**
PC 32:2	3.35±0.94	3.52±0.80	4,25±0.70	5.61±1.27	**9.32×10^{-10}**	4.88	9.19×10^{-1}	31.92	**1.98×10^{-4}**
PC 32:1	6.55±2.15	6.79±1.71	8.41±2.11	13.26±4.49	**6.10×10^{-10}**	3.72	9.03×10^{-1}	57.56	**1.29×10^{-4}**
PC32:0	6.93±1.03	5.80±0.85	6.75±1.35	8.16±1.49	**2.25×10^{-7}**	-16.32	**4.91×10^{-4}**	20.87	**4.40×10^{-3}**
PC 33:3	0.02±0.01	0.02±0.01	0.02±0.01	0.02±0.01	**6.31×10^{-3}**	-4.50	1.00×10^{0}	12.30	4.51×10^{-3}
PC 33:2	1.36±0.44	1.57±0.41	1.28±0.34	1.65±0.31	**1.99×10^{-3}**	-6.05	9.99×10^{-1}	5.31	9.60×10^{-3}
PC 33:1	1.11±0.28	1.14±0.31	1.02±0.34	1.42±0.49	**5.96×10^{-3}**	-7.65	8.57×10^{-1}	24.98	1.51×10^{-3}
PC 33:0	0.64±0.16	0.61±0.21	0.59±0.23	0.71±0.26	1.15×10^{-1}	-7.80	6.40×10^{-1}	16.40	3.21×10^{-1}
PC 34:5	0.01±0.01	0.01±0.01	0.02±0.01	0.03±0.02	**2.17×10^{-5}**	11.00	9.91×10^{-1}	87.67	9.30×10^{-2}
PC 34:4	0.46±0.21	0.5±0.19	0.68±0.21	0.95±0.32	**7.48×10^{-8}**	8.57	9.32×10^{-1}	38.48	**2.15×10^{-2}**
PC 34:3	6,57±1.95	6.05±1.23	7.68±1.68	10.19±2.6	**3.78×10^{-8}**	-7.92	9.99×10^{-1}	32.68	**2.48×10^{-3}**
PC 34:2	247.63±30.8	225.89±32.84	250.84±35.08	274.68±38.6	**5.42×10^{-4}**	-8.78	1.74×10^{-1}	9.50	1.66×10^{-3}
PC 34:1	101.81±19.19	91±15.14	114.39±18.32	136.79±27.09	**1.35×10^{-8}**	-10.61	8.83×10^{-1}	19.58	**1.57×10^{-2}**
PC 34:0	1.76±0.26	1.55±0.36	2.23±0.47	2.45±0.42	**5.60×10^{-10}**	-12.41	**3.68×10^{-1}**	10.21	3.84×10^{-1}

PC 35:4	0.45±0.15	0.53±0.17	0.45±0.17	0.59±0.14	**3.51×10^{-3}**	−0.81	1.00×10^{0}	11.59	7.77×10^{-1}
PC 35:3	0.62±0.20	0.71±0.18	0.57±0.15	0.74±0.13	**8.96×10^{-4}**	−7.62	9.99×10^{-1}	4.13	7.77×10^{-1}
PC 35:2	5.11±1.21	5.55±1.67	4.63±1.25	5.14±0.78	2.03×10^{-1}	−9.45	6.69×10^{-1}	−7.38	9.98×10^{-1}
PC 35:1	2.18±0.56	2.40±0.69	2.09±0.53	2.70±0.74	**4.25×10^{-3}**	−4.21	9.99×10^{-1}	12.33	3.45×10^{-1}
PC 35:0	0.07±0.03	0.09±0.05	0.07±0.04	0.12±0.07	**6.62×10^{-3}**	−1.16	1.00×10^{0}	29.59	4.51×10^{-1}
PC 36:6	0.22±0.10	0.24±0.10	0.34±0.12	0.43±0.14	**2.53×10^{-7}**	10.98	8.36×10^{-1}	24.77	1.51×10^{-3}
PC 36:5	5,46±2.30	5.39±1.62	6.92±2.39	9.93±3.81	**2.09×10^{-6}**	−1.33	9.97×10^{-1}	43.48	**1.90×10^{-3}**
PC 36:3	83.21±13.6	76.68±11.71	92.02±12.33	107.5±13.77	**5.71×10^{-9}**	−7.85	6.11×10^{-1}	16.83	**1.59×10^{-3}**
PC 36:2	189.08±24.63	174.54±28.51	202.01±36.07	219.53±34.27	**1.06×10^{-4}**	−7.69	2.55×10^{-1}	8.68	2.87×10^{-1}
PC 36:1	30.47±6.26	29.33±5.13	37.38±9.09	49.46±12.84	**4.19×10^{-9}**	−3.76	9.64×10^{-1}	32.31	**2.14×10^{-3}**
PC 36:0	0.16±0.04	0.16±0.06	0.23±0.07	0.32±0.23	**4.12×10^{-7}**	−2.90	9.39×10^{-1}	38.02	3.58×10^{-1}
PC 37:6	0.16±0.08	0.23±0.09	0.17±0.08	0.23±0.08	**2.42×10^{-4}**	3.68	1.00×10^{0}	0.15	1.00×10^{0}
PC 37:5	0.22±0.09	0.21±0.08	0.27±0.11	0.29±0.12	**2.59×10^{-2}**	−5.93	9.93×10^{-1}	8.39	9.98×10^{-1}
PC 37:4	3.19±0.76	3.31±0.85	3.66±1.01	3.62±0.73	1.18×10^{-1}	3.61	9.94×10^{-1}	−1.09	1.00×10^{0}
PC 38:7	0.72±0.31	0.66±0.22	0.98±0.34	0.99±0.20	**5.63×10^{-6}**	−8.83	9.96×10^{-1}	1.78	9.79×10^{-1}
PC 38:5	34.27±5.06	32.29±5.78	40.97±6.74	44.11±6.23	**2.24×10^{-8}**	−5.80	7.65×10^{-1}	7.65	1.74×10^{-1}
PC 38:4	77.61±13.76	81.63±17.48	96.57±20.19	103.97±16.77	**4.11×10^{-6}**	5.19	9.60×10^{-1}	7.67	6.26×10^{-3}
PC 38:3	27.42±7.29	29.87±8.61	38.12±10.35	54.11±13.02	**1.89×10^{-10}**	8.92	9.19×10^{-1}	41.92	**2.54×10^{-3}**
PC 38:2	11,56±2.72	10.28±2.14	12.56±2.39	15.08±3.34	**6.66×10^{-6}**	−11.08	5.08×10^{-1}	20.10	**2.91×10^{-2}**
PC 39:7	0.03±0.02	0.03±0.01	0.03±0.02	0.02±0.01	4.23×10^{-1}	7.64	9.97×10^{-1}	−21.71	4.37×10^{-3}
PC 39:6	0.69±0.30	0.77±0.30	0.66±0.25	0.71±0.21	4.44×10^{-1}	−3.31	1.00×10^{0}	−8.16	9.90×10^{-3}
PC 39:5	0.48±0.13	0.59±0.17	0.49±0.13	0.58±0.13	**1.13×10^{-2}**	1.79	1.00×10^{0}	−1.63	1.00×10^{0}

PC 40:8	0.92±0.30	0.74±0.21	1.05±0.33	0.91±0.18	**1.59×10^{-3}**	-19.12	1.03×10^{-1}	-13.45	6.26×10^{-1}
PC 40:7	2.58±0.84	2.23±0.59	3.09±0.95	2.79±0.55	**1.50×10^{-3}**	-13.62	7.65×10^{-1}	-9.83	9.84×10^{-1}
PC 40:6	15.93±4.14	18.12±4.57	21.77±6.8	27.1±6.52	**8.52×10^{-8}**	13.74	2.55×10^{-1}	24.52	**3.47×10^{-2}**
PC 40:5	10.52±2.23	10.83±2.33	14.26±3.60	18.53±4.5	**6.02×10^{-10}**	2.90	9.99×10^{-1}	29.87	**8.18×10^{-3}**
PC 40:4	3.88±1.07	3.80±1.11	5.15±1.39	6.64±1.62	**7.02×10^{-7}**	-2.01	9.99×10^{-1}	28.85	**1.14×10^{-2}**
Total PC(O)	53.49±10.56	51.64±13.01	51.17±8.88	49.65±7.97	6.95×10^{-1}	-3.46	4.20×10^{-1}	-2.96	5.20×10^{-1}
PC(O-32:2)	0.02±0.02	0.02±0.02	0.03±0.03	0.02±0.01	9.84×10^{-1}	-1.69	1.00×10^{0}	-17.92	1.00×10^{0}
PC(O-32:1)	0.26±0.07	0.26±0.08	0.25±0.06	0.25±0.07	9.87×10^{-1}	1.28	1.00×10^{0}	1.49	1.00×10^{0}
PC (O-32:0)	1.60±0.34	1.49±0.36	1.47±0.33	1.60±0.40	3.92×10^{-1}	-6.91	6.40×10^{-1}	9.15	8.86×10^{-1}
PC(O-34:4)	0.11±0.04	0.11±0.04	0.11±0.05	0.11±0.04	9.47×10^{-1}	3.45	1.00×10^{0}	0.72	9.86×10^{-3}
PC(O-34:3)	0.07±0.03	0.08±0.04	0.07±0.04	0.06±0.03	6.21×10^{-1}	0.80	1.00×10^{0}	-11.49	1.00×10^{0}
PC(O-34:2)	4.62±1.28	4.09±1.43	4.10±1.15	3.47±0.82	**2.10×10^{-2}**	-11.49	5.82×10^{-1}	-15.25	3.21×10^{-1}
PC(O-34:1)	3.10±0.70	2.79±0.77	2.57±0.73	2.69±0.62	**4.04×10^{-2}**	-10.03	4.37×10^{-1}	4.78	9.60×10^{-1}
PC(O-35:4)	0.08±0.04	0.08±0.05	0.08±0.05	0.08±0.04	9.62×10^{-1}	4.89	1.00×10^{0}	-1.14	1.00×10^{0}
PC(O-36:5)	0.36±0.22	0.32±0.18	0.34±0.15	0.33±0.15	9.66×10^{-1}	-11.11	9.97×10^{-1}	-3.40	9.97×10^{-1}
PC(O-36:4)	12.84±3.03	12.59±3.48	12.78±2.48	12.29±1.9	9.28×10^{-1}	-1.92	9.98×10^{-1}	-3.88	9.98×10^{-1}
PC(O-36:3)	4.41±1.08	4.20±1.20	4.12±0.67	3.91±0.72	4.54×10^{-1}	-4.93	9.55×10^{-1}	-5.03	8.25×10^{-1}
PC(O-36:2)	2.09±0.50	1.86±0.54	1.89±0.45	1.68±0.35	**4.54×10^{-2}**	-10.85	5.38×10^{-1}	-10.64	6.26×10^{-1}
PC(O-36:1)	0.27±0.12	0.26±0.10	0.22±0.08	0.26±0.11	5.52×10^{-1}	-2.54	1.00×10^{0}	14.77	8.67×10^{-1}
PC (O-36:0)	0.03±0.01	0.02±0.01	0.02±0.01	0.03±0.01	2.96×10^{-7}	-8.55	9.11×10^{-1}	15.14	4.94×10^{-1}
PC(O-38:5)	10.11±1.95	10.06±2.5	9.75±1.79	9.49±1.73	7.57×10^{-1}	-0.53	1.00×10^{0}	-2.68	9.99×10^{-1}
PC(O-38:4)	7.67±1.67	2.59±1.02	7.62±1.67	7.68±1.37	8.76×10^{-1}	-2.62	9.81×10^{-1}	0.86	1.00×10^{0}
PC(O-40:7)	1.53±0.37	1.60±0.5	1.53±0.43	1.43±0.25	7.65×10^{-1}	4.60	1.00×10^{0}	-6.94	9.98×10^{-1}

PC(O-40:6)	1.43±0.38	1.46±0.32	0.54±0.16	0.44±0.10	3.63×10^{-1}	2.02	1.00×10^{0}	-1.13	9.99×10^{-1}
PC(O-40:5)	2.89±0.58	2.88±0.80	2.88±0.58	2.94±0.54	8.92×10^{-1}	-0.51	9.96×10^{-1}	2.14	1.00×10^{0}
Total PC(P)	27.19±5.59	27.32±7.22	26.93±5.35	25.71±3.36	8.75×10^{-1}	0.50	1.00×10^{0}	-4.60	9.99×10^{-1}
PC(P-32:1)	0.15±0.04	0.16±0.05	0.13±0.03	0.14±0.03	3.68×10^{-1}	4.48	1.00×10^{0}	7.79	9.68×10^{-1}
PC (P-32:0)	1.13±0.24	1.10±0.27	1.08±0.22	1.13±0.24	9.14×10^{-1}	-2.43	9.88×10^{-1}	5.03	9.93×10^{-1}
PC(P-34:3)	0.02±0.01	0.02±0.004	0.02±0.005	0.02±0.01	**1.17×10^{-2}**	-10.68	8.67×10^{-1}	23.25	5.17×10^{-1}
PC(P-34:2)	5.81±1.26	5.31±1.32	5.21±1.13	4.85±1.05	6.55×10^{-2}	-8.60	5.23×10^{-1}	-7.04	9.03×10^{-1}
PC(P-34:1)	1.70±0.34	1.62±0.37	1.54±0.33	1.44±0.29	7.19×10^{-2}	-4.69	9.86×10^{-1}	-6.55	8.95×10^{-1}
PC(P-36:5)	0.16±0.09	0.18±0.09	0.16±0.08	0.21±0.10	6.07×10^{-2}	8.53	9.90×10^{-1}	28.78	2.98×10^{-1}
PC(P-36:4)	7.49±1.60	7.84±2.10	7.68±1.71	7.55±1.03	9.93×10^{-1}	4.63	9.99×10^{-1}	-1.70	1.00×10^{0}
PC(P-36:2)	1.83±0.49	1.75±0.60	1.71±0.47	1.50±0.28	2.01×10^{-1}	-4.74	9.39×10^{-1}	-12.61	4.65×10^{-1}
PC(P-38:6)	0.49±0.15	0.55±0.24	0.52±0.16	0.48±0.10	9.87×10^{-1}	10.95	1.00×10^{0}	-6.13	9.99×10^{-1}
PC(P-38:5)	3.78±0.84	4.05±1.12	3.90±0.80	3.75±0.46	9.87×10^{-1}	7.05	1.00×10^{0}	-3.80	9.99×10^{-1}
PC(P-38:4)	2.42±0.66	2.59±1.02	2.68±0.81	2.52±0.43	7.65×10^{-1}	6.83	9.96×10^{-1}	-5.83	1.00×10^{0}
PC(P-40:6)	0.52±0.19	0.52±0.24	0.54±0.16	0.44±0.10	3.41×10^{-1}	0.81	1.00×10^{0}	-19.47	1.00×10^{0}
PC(P-40:5)	1.62±0.42	1.59±0.62	1.71±0.49	1.62±0.26	6.95×10^{-1}	-1.86	1.00×10^{0}	-5.24	2.45×10^{-1}
Total LPC	155.74±19.72	129.75±24.39	174.57±23.75	168.49±25.28	8.07×10^{-7}	-16.69	2.67×10^{-3}	-3.48	9.75×10^{-1}
LPC 14:0	0.95±0.48	0.95±0.31	1.37±0.46	1.61±0.51	1.55×10^{-6}	-0.43	9.88×10^{-1}	17.15	5.67×10^{-1}
LPC 15:0	0.54±0.17	0.50±0.13	0.68±0.17	0.61±0.13	7.07×10^{-4}	-8.17	9.91×10^{-1}	-10.07	5.23×10^{-1}
LPC 16:1	1.83±0.45	1.72±0.49	2.33±0.51	2.53±0.57	1.88×10^{-6}	-6.24	9.84×10^{-1}	8.91	7.39×10^{-1}
LPC 16:0	83.14±10.78	72.87±12.73	99.19±14.89	99.7±16.66	2.06×10^{-8}	-12.35	3.27×10^{-2}	0.51	1.00×10^{0}
LPC 17:1	0.24±0.05	0.21±0.05	0.29±0.06	0.28±0.07	2.17×10^{-5}	-12.07	6.44×10^{-2}	-5.01	9.03×10^{-1}

LPC 17:0	0.91±0.22	0.82±0.26	1.12±0.31	0.94±0.24	1.45×10^{-3}	-10.17	5.08×10^{-1}	-16.41	9.30×10^{-2}
LPC 18:3	0.38±0.15	0.28±0.11	0.36±0.12	0.37±0.11	2.16×10^{-2}	-25.82	6.79×10^{-2}	3.92	9.98×10^{-1}
LPC 18:2	28.16±6.79	19.35±5.84	24±5.6	19.61±3.48	**8.07×10^{-7}**	-31.29	**1.08×10^{-4}**	-18.14	**2.02×10^{-2}**
LPC 18:1	13.66±2.53	10.11±2.4	13.69±2.51	11.76±1.69	**2.39×10^{-6}**	-26.01	**9.91×10^{-5}**	-14.13	**2.02×10^{-2}**
LPC 18:0	18.25±2.74	16.23±3.66	22.82±3.99	22.77±4.36	**5.54×10^{-8}**	-11.07	1.58×10^{-1}	-0.19	9.98×10^{-1}
LPC 20:5	0.17±0.08	0.15±0.07	0.19±0.09	0.23±0.09	**8.52×10^{-4}**	-12.77	8.47×10^{-1}	19.39	5.82×10^{-1}
LPC 20:4	4.20±0.70	3.68±1.04	4.71±1.08	4.27±0.94	**1.13×10^{-2}**	-12.38	1.66×10^{-1}	-9.36	5.23×10^{-1}
LPC 20:3	1.46±0.34	1.29±0.34	1.7±0.29	1.86±0.39	**3.00×10^{-6}**	-11.49	3.09×10^{-1}	9.42	6.11×10^{-1}
LPC 20:2	0.22±0.05	0.17±0.04	0.23±0.04	0.2±0.05	**7.58×10^{-5}**	-23.15	**4.40×10^{-3}**	-14.69	**3.47×10^{-2}**
LPC 20:1	0.16±0.05	0.12±0.03	0.16±0.04	0.13±0.03	**7.28×10^{-5}**	-22.88	**6.67×10^{-3}**	-22.27	**4.40×10^{-3}**
LPC 20:0	0.09±0.03	0.07±0.02	0.1±0.02	0.08±0.02	**1.33×10^{-5}**	-27.66	**7.33×10^{-4}**	-18.36	**2.28×10^{-2}**
LPC 22:6	0.88±0.18	0.83±0.3	1.1±0.35	1.01±0.20	**3.22×10^{-3}**	-5.46	8.02×10^{-1}	-8.28	1.00×10^{0}
LPC 22:5	0.39±0.06	0.32±0.08	0.47±0.1	0.46±0.08	**3.02×10^{-6}**	-16.89	**4.13×10^{-2}**	-2.17	1.00×10^{0}
LPC 22:1	0.01±0.01	0.01±0.002	0.01±0.01	0.01±0.002	**1.32×10^{-3}**	-24.53	8.39×10^{-2}	-30.10	**1.38×10^{-2}**
LPC 22:0	0.03±0.01	0.02±0.01	0.02±0.01	0.02±0.01	1.61×10^{-1}	-20.98	4.24×10^{-1}	-12.98	7.25×10^{-1}
LPC 24:0	0.06±0.02	0.04±0.01	0.06±0.02	0.05±0.01	**3.73×10^{-3}**	-21.73	**3.27×10^{-2}**	-6.68	9.26×10^{-1}
LPC 26:0	0.01±0.004	0.01±0.003	0.01±0.01	0.01±0.004	**5.39×10^{-3}**	-24.44	**1.14×10^{-1}**	-16.77	3.45×10^{-1}
Total PE	13.56±4.44	12.35±5.19	21.36±5.15	23.01±8.22	**2.21×10^{-9}**	-8.89	3.10×10^{-1}	7.72	4.70×10^{-1}
PE 34:3	0.04±0.02	0.03±0.02	0.05±0.03	0.07±0.04	**4.32×10^{-5}**	-16.35	8.67×10^{-1}	42.11	1.31×10^{-1}
PE 34:2	0.88±0.27	0.75±0.35	1.23±0.46	1.49±0.63	**2.10×10^{-7}**	-14.76	5.23×10^{-1}	20.82	2.87×10^{-1}
PE 34:1	0.33±0.12	0.27±0.11	0.45±0.19	0.59±0.25	**9.51×10^{-8}**	-17.05	3.33×10^{-1}	33.25	**3.47×10^{-2}**
PE 35:2	0.05±0.02	0.04±0.02	0.06±0.02	0.06±0.03	**1.82×10^{-2}**	-13.92	9.11×10^{-1}	-3.04	9.72×10^{-1}
PE 35:1	0.04±0.02	0.03±0.02	0.04±0.02	0.05±0.03	**7.86×10^{-3}**	-25.21	2.55×10^{-1}	13.76	9.32×10^{-1}

PE 36:5	0.04±0.02	0.02±0.01	0.04±0.02	0.06±0.04	**4.05×10^{-4}**	-32.17	3.33×10^{-1}	37.93	5.38×10^{-1}
PE 36:4	1.13±0.33	1.01±0.43	1.62±0.35	1.64±0.57	**8.49×10^{-7}**	-10.28	7.39×10^{-1}	1.41	1.00×10^{0}
PE 36:3	0.77±0.25	0.59±0.23	1.01±0.34	0.99±0.40	**1.22×10^{-5}**	-23.64	6.44×10^{-2}	-2.59	9.94×10^{-1}
PE 36:2	2.76±0.93	2.41±1.00	4.24±1.45	5.06±2.26	**6.60×10^{-9}**	-12.65	8.14×10^{-1}	19.36	3.97×10^{-1}
PE 36:1	0.41±0.12	0.37±0.13	0.62±0.3	0.85±0.51	**3.09×10^{-9}**	-10.53	8.86×10^{-1}	36.86	1.44×10^{-1}
PE 38:6	1.36±0.65	1.29±0.72	2.16±0.63	21.9±0.94	**1.95×10^{-6}**	-4.97	9.98×10^{-1}	1.38	1.00×10^{0}
PE 38:5	0.92±0.38	0.76±0.34	1.38±0.35	1.28±0.47	**2.09×10^{-6}**	-17.25	7.65×10^{-1}	-7.03	8.25×10^{-1}
PE 38:4	3.17±1.07	3.05±1.36	5.28±1.11	5.13±1.67	**1.46×10^{-8}**	-3.66	9.96×10^{-1}	-2.88	9.81×10^{-1}
PE 38:3	0.62±0.22	0.61±0.28	1.07±0.27	1.16±0.43	**1.60×10^{-9}**	-1.95	9.94×10^{-1}	8.67	9.39×10^{-1}
PE 40:7	0.12±0.07	0.11±0.06	0.18±0.06	0.15±0.06	**1.25×10^{-4}**	-12.87	9.45×10^{-1}	-17.06	3.58×10^{-1}
PE 40:6	0.65±0.33	0.73±0.36	1.37±0.54	1.58±0.63	**1.11×10^{-9}**	11.69	9.79×10^{-1}	14.94	7.90×10^{-1}
PE 40:5	0.29±0.13	0.28±0.15	0.57±0.2	0.68±0.29	**6.44×10^{-10}**	-0.19	1.00×10^{0}	19.53	6.97×10^{-1}
Total PE(O)	3.08±0.98	2.74±1.05	3.4±0.85	2.81±0.56	**3.20×10^{-2}**	-11.10	1.50×10^{-1}	-17.18	**2.00×10^{-2}**
PE(O-34:2)	0.11±0.04	0.09±0.04	0.12±0.04	0.09±0.03	**1.78×10^{-2}**	-20.52	2.45×10^{-1}	-24.31	6.79×10^{-1}
PE(O-34:1)	0.13±0.05	0.11±0.04	0.14±0.05	0.11±0.03	1.34×10^{-1}	-14.61	7.25×10^{-1}	-18.90	2.55×10^{-1}
PE(O-36:6)	0.02±0.01	0.02±0.01	0.02±0.02	0.03±0.02	1.96×10^{-1}	-19.03	9.32×10^{-1}	19.88	7.39×10^{-1}
PE(O-36:5)	0.03±0.01	0.02±0.02	0.04±0.03	0.03±0.02	1.90×10^{-1}	-19.60	4.10×10^{-1}	-23.66	9.86×10^{-1}
PE(O-36:4)	0.76±0.24	0.85±0.24	0.70±0.33	0.66±0.17	**2.18×10^{-2}**	-8.57	5.52×10^{-1}	-22.43	**1.78×10^{-2}**
PE(O-36:3)	0.17±0.05	0.18±0.05	0.14±0.06	0.14±0.04	**2.13×10^{-2}**	-15.73	3.45×10^{-1}	-20.22	9.30×10^{-1}
PE(O-36:2)	0.16±0.06	0.14±0.05	0.18±0.06	0.15±0.04	1.15×10^{-1}	-15.39	6.69×10^{-1}	-16.91	4.37×10^{-1}
PE(O-38:5)	0.61±0.20	0.66±0.17	0.54±0.21	0.57±0.13	1.49×10^{-1}	-11.09	9.11×10^{-1}	-13.93	3.45×10^{-1}
PE(O-38:4)	0.73±0.33	0.82±0.29	0.65±0.30	0.66±0.18	1.51×10^{-1}	-10.52	9.50×10^{-1}	-16.61	6.40×10^{-1}
PE(O-40:7)	0.16±0.06	0.16±0.05	0.16±0.05	0.14±0.03	5.41×10^{-1}	-4.11	1.00×10^{0}	-9.94	7.52×10^{-1}

PE(O-40:6)	0.08±0.02	0.06±0.03	0.08±0.02	0.08±0.03	**1.90×10^{-2}**	-17.09	1.25×10^{-1}	-9.55	8.77×10^{-1}
PE(O-40:5)	0.13±0.05	0.16±0.05	0.12±0.05	0.15±0.05	**8.87×10^{-3}**	-7.99	9.50×10^{-1}	-10.21	8.14×10^{-1}
Total PE(P)	3.60±1.13	3.90±1.02	3.59±1.11	3.68±0.63	3.78×10^{-1}	-0.45	1.00×10^{0}	-5.63	9.93×10^{-1}
PE(P-34:2)	0.14±0.05	0.13±0.04	0.13±0.04	0.13±0.04	9.28×10^{-1}	-2.64	9.99×10^{-1}	-4.09	1.00×10^{0}
PE(P-34:1)	0.07±0.02	0.06±0.01	0.06±0.03	0.06±0.02	6.16×10^{-1}	-11.34	6.97×10^{-1}	-2.73	9.99×10^{-1}
PE(P-36:4)	0.47±0.19	0.47±0.16	0.48±0.18	0.49±0.10	4.08×10^{-1}	1.85	9.98×10^{-1}	4.01	7.52×10^{-1}
PE(P-36:2)	0.29±0.11	0.30±0.09	0.27±0.10	0.27±0.08	6.09×10^{-1}	-8.49	9.75×10^{-1}	-9.87	9.26×10^{-1}
PE(P-36:1)	0.05±0.02	0.05±0.03	0.05±0.02	0.06±0.01	5.52×10^{-1}	1.07	1.00×10^{0}	1.76	9.72×10^{-1}
PE(P-38:6)	0.25±0.07	0.27±0.09	0.27±0.09	0.29±0.06	1.53×10^{-1}	7.55	9.93×10^{-1}	8.20	6.40×10^{-1}
PE(P-38:5)	0.98±0.29	1.09±0.27	0.98±0.29	0.98±0.15	3.05×10^{-1}	-0.36	1.00×10^{0}	-9.30	7.25×10^{-1}
PE(P-38:4)	0.74±0.32	0.83±0.28	0.77±0.30	0.77±0.18	3.78×10^{-1}	3.20	9.94×10^{-1}	-7.22	9.97×10^{-1}
PE(P-40:6)	0.22±0.08	0.24±0.09	0.22±0.08	0.24±0.06	4.33×10^{-1}	-2.68	9.94×10^{-1}	-2.18	1.00×10^{0}
PE(P-40:5)	0.33±0.10	0.39±0.12	0.32±0.10	0.34±0.09	9.19×10^{-1}	-4.98	9.95×10^{-1}	-11.32	7.11×10^{-1}
PE(P-40:4)	0.05±0.03	0.06±0.03	0.05±0.02	0.04±0.03	2.64×10^{-1}	-12.10	9.99×10^{-1}	-26.18	2.16×10^{-1}
Total LPE	14.22±3.22	10.12±3.13	14.58±2.8	13.56±2.53	6.81×10^{-6}	-28.84	2.77×10^{-4}	-7.02	8.57×10^{-1}
LPE 16:0	1.92±0.48	2.17±0.49	1.42±0.43	2.05±0.43	5.09×10^{-6}	-26.02	**4.40×10^{-3}**	-5.56	9.93×10^{-1}
LPE 18:2	4.99±1.54	4.36±1.28	3.23±1.33	4.12±1.22	7.96×10^{-5}	-35.18	**3.01×10^{-4}**	-5.51	9.81×10^{-1}
LPE 18:1	2.45±0.76	2.19±0.67	1.51±0.69	2.06±0.67	**6.81×10^{-6}**	-38.28	**1.18×10^{-4}**	-5.95	9.32×10^{-1}
LPE 18:0	2.72±0.61	3.48±0.62	2.13±0.56	3.22±0.61	**1.18×10^{-8}**	-21.84	**9.98×10^{-3}**	-7.47	7.65×10^{-1}
LPE 20:4	1.33±0.32	1.42±0.24	1.10±0.30	1.21±0.18	**7.05×10^{-4}**	-17.65	7.96×10^{-2}	-14.47	9.34×10^{-3}
LPE 22:6	0.81±0.15	0.96±0.31	0.73±0.25	0.90±0.19	**1.62×10^{-2}**	-9.76	6.62×10^{-1}	-6.99	1.00×10^{0}
Total PI	96.47±14.25	89.48±20.28	125.41±28.25	143.31±21.89	**1.62×10^{-10}**	-7.24	1.10×10^{-1}	14.27	**6.00×10^{-3}**

PI 32:1	0.48±0.28	0.51±0.22	0.74±0.49	1.82±1.11	**2.73×10^{-10}**	6.66	9.55×10^{-1}	147.11	**8.14×10^{-6}**
PI 32:0	0.34±0.14	0.31±0.12	0.47±0.26	1.01±0.59	**5.71×10^{-9}**	-9.40	9.39×10^{-1}	112.29	**3.85×10^{-1}**
PI 34:1	6.24±1.57	5.16±1.48	7.97±2.75	10.8±3.22	**9.79×10^{-10}**	-17.29	5.17×10^{-2}	35.39	**7.64×10^{-1}**
PI 34:0	0.13±0.06	0.1±0.05	0.17±0.07	0.28±0.12	**4.26×10^{-8}**	-18.36	8.36×10^{-1}	66.00	**3.08×10^{-1}**
PI 36:4	6.60±1.80	5.93±1.86	9.13±2.33	10.98±2.67	**2.35×10^{-10}**	-10.19	5.82×10^{-1}	20.25	6.44×10^{-2}
PI 36:3	6.03±1.39	4.65±1.77	7.09±2.36	7.15±1.53	**8.21×10^{-7}**	-22.98	**2.48×10^{-3}**	0.89	9.96×10^{-1}
PI 36:2	24.52±4.37	22.55±6.01	29.78±8.61	33.92±6.54	**1.49×10^{-7}**	-8.03	6.26×10^{-1}	13.91	1.03×10^{-1}
PI 36:1	5.78±1.60	4.71±1.75	6.86±2.7	7.62±2.16	**2.63×10^{-5}**	-18.60	**3.47×10^{-2}**	11.12	7.52×10^{-1}
PI 38:6	0.86±0.30	0.71±0.30	1.06±0.39	1.11±0.28	**3.11×10^{-5}**	-17.80	2.07×10^{-1}	4.72	8.67×10^{-1}
PI 38:5	2.28±0.54	1.8±0.46	2.73±0.85	2.84±0.63	**1.46×10^{-6}**	-21.06	**1.21×10^{-2}**	4.29	9.72×10^{-1}
PI 38:4	33.54±4.83	33.67±7.55	46.11±9.64	50.02±8.96	**3.73×10^{-10}**	0.40	9.99×10^{-1}	8.49	5.82×10^{-1}
PI 38:3	6.62±2.14	6.42±1.61	9.01±1.98	11±2.73	**4.73×10^{-10}**	-3.11	1.00×10^{0}	22.14	**1.30×10^{-2}**
PI 38:2	0.42±0.15	0.39±0.14	0.61±0.17	0.55±0.17	**1.08×10^{-5}**	-7.79	9.88×10^{-1}	-9.77	8.02×10^{-1}
PI 40:6	1.21±0.37	1.21±0.42	1.69±0.17	1.79±0.45	**5.29×10^{-6}**	-0.56	1.00×10^{0}	6.04	8.77×10^{-1}
PI 40:5	0.95±0.19	0.94±0.24	1.34±0.35	1.68±0.47	**8.23×10^{-10}**	-1.16	9.91×10^{-1}	25.18	6.44×10^{-1}
PI 40:4	0.46±0.13	0.43±0.13	0.66±0.15	0.73±0.17	**1.65×10^{-9}**	-5.79	8.14×10^{-1}	11.13	5.82×10^{-1}
Total LPI	3.48±0.77	3.21±0.58	3.99±0.82	4.59±1.14	**4.17×10^{-6}**	-7.53	1.70×10^{-1}	15.12	**4.70×10^{-2}**
LPI 18:2	1.07±0.30	1.09±0.32	0.91±0.24	1.34±0.45	**1.87×10^{-3}**	-15.55	3.21×10^{-1}	23.06	1.98×10^{-1}
LPI 18:1	0.52±0.16	0.57±0.23	0.45±0.13	0.66±0.35	**3.96×10^{-2}**	-14.42	4.51×10^{-1}	15.14	9.84×10^{-1}
LPI 18:0	0.85±0.29	1.14±0.24	0.90±0.19	1.26±0.29	**1.16×10^{-6}**	5.02	9.50×10^{-1}	10.30	5.96×10^{-1}
LPI 20:4	1.03±0.29	1.19±0.27	0.97±0.22	1.34±0.38	**3.15×10^{-4}**	-6.09	9.84×10^{-1}	12.44	4.80×10^{-1}
Total PS	0.63±0.24	0.62±0.22	0.64±0.23	0.65±0.24	9.84×10^{-1}	-1.24	9.90×10^{-1}	1.66	7.60×10^{-1}
PS 36:2	0.04±0.01	0.04±0.02	0.04±0.02	0.05±0.02	**4.38×10^{-2}**	6.24	9.90×10^{-1}	17.58	6.26×10^{-1}

PS 36:1	0.24±0.10	0.23±0.08	0.24±0.09	0.24±0.09	9.87×10^{-1}	-2.79	1.00×10^{0}	2.92	1.00×10^{0}
PS 38:4	0.23±0.10	0.22±0.10	0.22±0.11	0.20±0.09	9.28×10^{-1}	-5.15	9.94×10^{-1}	-8.90	9.99×10^{-1}
PS 38:3	0.06±0.03	0.06±0.02	0.06±0.03	0.07±0.03	7.32×10^{-1}	-5.06	9.72×10^{-1}	16.28	9.03×10^{-1}
PS 40:6	0.04±0.02	0.04±0.02	0.04±0.02	0.04±0.03	6.24×10^{-1}	24.28	8.36×10^{-1}	16.40	9.68×10^{-1}
PS 40:5	0.03±0.02	0.03±0.02	0.04±0.02	0.04±0.03	2.98×10^{-1}	10.25	1.00×10^{0}	0.14	1.00×10^{0}
Total PG	0.09±0.03	0.14±0.05	0.07±0.05	0.19±0.09	**3.95×10^{-8}**	-18.35	3.97×10^{-1}	31.65	5.96×10^{-1}
PG 34:1	0.02±0.02	0.04±0.02	0.02±0.02	0.04±0.02	**8.90×10^{-6}**	4.53	1.00×10^{0}	-13.17	9.32×10^{-1}
PG 36:2	0.04±0.02	0.05±0.03	0.03±0.03	0.08±0.04	**2.78×10^{-5}**	-14.28	7.65×10^{-1}	46.81	1.51×10^{-1}
PG 36:1	0.03±0.01	0.02±0.02	0.05±0.03	0.07±0.04	**5.98×10^{-8}**	-38.44	**9.93×10^{-3}**	55.65	1.25×10^{-1}
Total CE	979.72± 110.87	961.45± 140.04	1197.35± 159.6	1481.38± 189.7	**1.79×10^{-12}**	-1.87	3.00×10^{-1}	23.72	**1.75×10^{-5}**
CE 14:0	5.96±2.32	5.69±1.88	8.1±2.47	11.9±5.30	**1.79×10^{-7}**	-4.53	1.00×10^{0}	46.94	**3.90×10^{-2}**
CE 15:0	4.70±1.60	4.5±1.37	5.85±1.55	6.95±2.52	**7.76×10^{-5}**	-4.16	1.00×10^{0}	18.95	5.67×10^{-1}
CE 16:2	1.13±0.24	1.16±0.30	1，76±0.46	2.75±0.76	**6.45×10^{-13}**	3.12	9.99×10^{-1}	56.36	**1.32×10^{-5}**
CE 16:1	36.49±14.36	38.21±11.09	47.89±13.95	80.49±32.52	**1.30×10^{-9}**	4.71	9.03×10^{-1}	68.07	**1.81×10^{-4}**
CE 16:0	344.8±34.05	345.4±50.85	423.25± 58.49	514.88±53.49	**3.12×10^{-12}**	0.18	1.00×10^{0}	21.65	**1.60×10^{-5}**
CE 17:1	16.53±2.46	16.19±2.67	19.68±2.71	24.74±3.21	**1.27×10^{-11}**	-2.08	9.84×10^{-1}	25.68	**1.09×10^{-5}**
CE 17:0	3.64±1.41	3.75±1.31	4.61±1.79	5.55±2.15	**4.57×10^{-4}**	2.97	9.91×10^{-1}	20.36	3.58×10^{-1}
CE 18:3	17.67±6.90	17.72±5.04	25.63±10.78	41.79±15.48	**8.85×10^{-11}**	0.27	9.97×10^{-1}	63.05	**2.54×10^{-4}**
CE 18:2	269.64±29.37	255.36± 31.99	298.94± 37.21	342.87±35.76	**9.76×10^{-11}**	-5.30	4.80×10^{-1}	14.70	**7.93×10^{-4}**
CE 18:1	172.07±29.37	159.88± 27.03	207.74± 34.43	252.73±49.58	**8.85×10^{-11}**	-7.09	4.37×10^{-1}	21.66	**5.82×10^{-3}**

CE 18:0	16.4±3.28	17.24±4.18	24.99±6.86	30.37±6.46	**2.22×10^{-11}**	5.11	9.79×10^{-1}	21.53	**4.37×10^{-2}**
CE 20:5	7.05±2.63	8.11±3.02	12.5±6.70	21.67±8.50	**1.05×10^{-10}**	15.03	6.26×10^{-1}	73.43	**3.01×10^{-4}**
CE 20:3	2.09±0.76	2.39±0.79	2.71±0.69	4.22±1.00	**3.30×10^{-10}**	14.20	3.97×10^{-1}	55.84	**1.63×10^{-6}**
CE 20:4	75.01±11.73	78.74±20.46	103.92±25.69	128.09±22.04	**8.21×10^{-11}**	4.98	9.72×10^{-1}	23.26	**1.07×10^{-2}**
CE 20:2	0.48±0.12	0.53±0.13	0.62±0.12	0.90±0.20	**5.07×10^{-11}**	11.06	3.97×10^{-1}	44.81	**6.69×10^{-6}**
CE 20:1	0.20±0.07	0.18±0.05	0.26±0.08	0.26±0.09	**1.35×10^{-4}**	-9.07	9.95×10^{-1}	-0.74	1.00×10^{0}
CE 22:6	5.09±1.21	5.64±1.46	7.84±2.76	9.93±2.70	**4.44×10^{-10}**	10.89	5.67×10^{-1}	26.53	**3.90×10^{-2}**
CE 22:5	0.41±0.08	0.4±0.09	0.54±0.12	0.70±0.16	**5.80×10^{-11}**	-1.67	1.00×10^{0}	28.45	**3.82×10^{-3}**
CE 22:4	0.05±0.01	0.05±0.01	0.06±0.01	0.07±0.01	**3.97×10^{-5}**	-6.19	9.79×10^{-1}	16.69	8.83×10^{-2}
CE 22:1	0.05±0.03	0.05±0.02	0.07±0.05	0.07±0.03	**5.60×10^{-3}**	-6.75	1.00×10^{0}	-3.84	1.00×10^{0}
CE 22:0	0.11±0.05	0.11±0.03	0.16±0.07	0.19±0.08	**2.19×10^{-6}**	-1.10	9.79×10^{-1}	20.40	3.21×10^{-1}
CE 24:6	0.003±0.001	0.003±0.001	0.004±0.002	0.01±0.002	**9.57×10^{-5}**	4.44	1.00×10^{0}	33.72	1.51×10^{-1}
CE 24:1	0.05±0.02	0.05±0.01	0.07±0.05	0.07±0.02	**5.31×10^{-5}**	-7.58	9.93×10^{-1}	4.36	1.00×10^{0}
CE 24:0	0.09±0.04	0.09±0.02	0.14±0.06	0.16±0.07	**1.82×10^{-8}**	-7.09	1.00×10^{0}	13.20	6.83×10^{-1}
COH	485.83±76.38	494.16±98.6	671.6±152.94	856.14±150.8	**1.61×10^{-11}**	1.71	9.99×10^{-1}	27.48	**2.48×10^{-3}**
Total DG	33.38±12.81	34.31±14.62	72.05±27.2	92.96±29.92	**1.01×10^{-12}**	2.78	9.20×10^{-1}	29.01	**9.00×10^{-3}**
DG 14:0/16:0	0.11±0.07	0.11±0.06	0.26±0.19	0.55±0.43	**2.20×10^{-10}**	-0.82	1.00×10^{0}	110.07	**7.14×10^{-3}**
DG 14:0/18:2	0.18±0.12	0.17±0.09	0.46±0.27	0.62±0.25	**1.53×10^{-11}**	-7.82	1.00×10^{0}	32.75	1.14×10^{-1}
DG 14:0/18:1	0.23±0.16	0.21±0.12	0.6±0.42	0.89±0.55	**1.10×10^{-10}**	-7.26	1.00×10^{0}	49.24	1.31×10^{-1}
DG 16:0/16:0	0.75±0.28	0.74±0.28	1.43±1.00	2.95±1.86	**7.40×10^{-12}**	-1.41	1.00×10^{0}	106.86	**2.15×10^{-4}**
DG 16:0/18:2	4.29±1.82	4.51±1.91	9.38±4.18	12.79±4.16	**1.74×10^{-12}**	4.99	9.79×10^{-1}	36.28	**1.38×10^{-2}**

DG 16:1/18:1	0.98±0.56	1.02±0.57	2.32±1.06	3.48±1.59	**2.54×10^{-12}**	3.49	1.00×10^{0}	50.00	**7.64×10^{-3}**
DG 16:0/18:1	4,51±1.97	4.73±2.27	9.84±5.01	14.84±6.70	**3.12×10^{-12}**	4.91	1.00×10^{0}	50.76	**5.07×10^{-3}**
DG 16:0/20:4	0.44±0.19	0.5±0.21	0.92±0.43	1.65±0.86	**4.34×10^{-12}**	12.79	8.47×10^{-1}	79.88	**4.91×10^{-4}**
DG 18:1/18:3	1.2±0.53	1.15±0.52	2.61±0.96	3.02±1.02	**1.33×10^{-11}**	-4.11	1.00×10^{0}	15.40	6.69×10^{-1}
DG 18:2/18:2	2.68±1.19	2.74±1.35	5.92±2.24	6.07±2.10	**1.33×10^{-10}**	2.19	1.00×10^{0}	2.48	9.99×10^{-1}
DG 16:0/20:3	0.14±0,06	0.16±0.07	0.31±0.15	0.46±0.19	**4.34×10^{-12}**	11.66	9.55×10^{-1}	48.90	**1.38×10^{-2}**
DG 18:1/18:2	8.58±3.51	8.6±3.94	18.55±5.95	20.45±5.48	**4.20×10^{-12}**	0.12	1.00×10^{0}	10.26	6.97×10^{-1}
DG 18:0/18:2	0.77±0.31	0.87±0.36	1.84±0.96	2.42±0.88	**1.79×10^{-12}**	11.85	9.39×10^{-1}	31.76	**3.90×10^{-2}**
DG 18:1/18:1	5.03±2.14	5.02±2.38	10.69±3.87	12.96±4.56	**7.72×10^{-12}**	-0.10	1.00×10^{0}	21.24	3.71×10^{-1}
DG 18:0/18:1	1.01±0.42	1.12±0.55	2.21±1.27	3.33±1.70	**1.37×10^{-11}**	11.19	9.96×10^{-1}	50.61	**1.47×10^{-2}**
DG 16:0/22:6	0.17±0.11	0.22±0.15	0.36±0.19	0.58±0.28	**1.14×10^{-9}**	29.69	7.25×10^{-1}	60.86	**1.07×10^{-2}**
DG 16:0/22:5	0.16±0.07	0.17±0.08	0.34±0.14	0.49±0.21	**1.12×10^{-11}**	9.35	9.75×10^{-1}	43.07	**4.62×10^{-2}**
DG 18:1/20:4	1.41±0.50	1.56±0.64	2.76±1.00	3.91±1.57	**1.37×10^{-11}**	9.79	9.93×10^{-1}	41.53	**2.15×10^{-2}**
DG 18:0/20:4	0.33±0.11	0.34±0.12	0.49±0.17	0.62±0.20	**5.98×10^{-8}**	3.73	1.00×10^{0}	27.04	1.38×10^{-1}
DG 18:1/20:3	0.39±0.16	0.38±0.17	0.77±0.29	0.91±0.30	**6.47×10^{-11}**	-2.09	1.00×10^{0}	17.84	5.08×10^{-1}
Total TG	157.56±60.78	162.15± 67.04	349.79± 142.61	490.86± 191.45	**6.45×10^{-13}**	2.91	8.50×10^{-1}	40.33	**2.00×10^{-3}**
TG 14:0/16:1/18:2	0.58±0.45	0.41±0.23	1.49±1.03	2.15±1.33	**6.45×10^{-11}**	-29.50	9.11×10^{-1}	44.41	1.74×10^{-1}
TG 16:1/16:1/16:1	0.12±0.10	0.11±0.07	0.34±0.22	0.55±0.32	**7.35×10^{-12}**	-7.98	1.00×10^{0}	60.79	**2.58×10^{-2}**
TG 14:0/16:0/18:2	1.18±1.06	1.02±0.6	2.97±2.54	5.99±4.14	**5.47×10^{-11}**	-14.01	1.00×10^{0}	102.15	**7.64×10^{-3}**

TG 14:0/16:1/18:1	1.06±0.92	0.74±0.43	2.54±1.79	4.54±4.53	**4.56×10^{-10}**	−29.60	9.79×10^{-1}	78.81	1.90×10^{-1}
TG 14:1/16:0/18:1	0.21±0.20	0.16±0.08	0.43±0.35	0.98±1.01	**3.26×10^{-10}**	−24.41	1.00×100	126.85	**2.28×10^{-2}**
TG 14:1/16:1/18:0	0.78±0.85	0.68±0.41	1.85±1.46	4.29±3.56	**4.07×10^{-11}**	−12.75	9.91×10^{-1}	132.09	**1.99×10^{-1}**
TG 14:0/16:0/18:1	1.68±1.53	1.41±0.92	4.24±3.41	8.52±6.66	**5.62×10^{-10}**	−15.80	1.00×10^{0}	100.78	**3.47×10^{-2}**
TG 16:0/16:0/16:0	0.84±0.91	0.66±0.46	1.6±1.94	5.06±4.25	**5.60×10^{-10}**	−21.60	9.94×10^{-1}	216.75	**2.77×10^{-4}**
TG 15:0/16:0/18:1	0.20±0.13	0.17±0.12	0.44±0.34	0.84±0.65	**2.21×10^{-9}**	−10.76	9.98×10^{-1}	88.73	**2.91×10^{-2}**
TG 16:0/16:1/17:0	0.40±0.27	0.41±0.28	0.88±0.69	1.87±1.76	**1.67×10^{-9}**	1.02	1.00×10^{0}	113.88	**9.98×10^{-3}**
TG 14:0/17:0/18:1	0.32±0.21	0.3±0.21	0.72±0.52	1.24±1.09	**4.72×10^{-9}**	−5.98	1.00×10^{0}	72.06	7.96×10^{-2}
TG 14:0/18:2/18:2	0.78±0.44	0.69±0.38	2.19±1.15	2.28±0.75	**4.72×10^{-12}**	−10.90	9.84×10^{-1}	4.42	9.60×10^{-1}
TG 14:1/18:0/18:2	0.08±0.04	0.07±0.03	0.17±0.08	0.22±0.13	**1.05×10^{-11}**	−10.86	9.90×10^{-1}	30.58	2.35×10^{-1}
TG 14:1/18:1/18:1	1.41±0.79	1.31±0.68	3.73±1.71	4.38±1.61	**3.22×10^{-12}**	−6.81	1.00×10^{0}	17.43	6.26×10^{-1}
TG 16:1/16:1/18:1	1.84±1.08	1.95±1.01	4.47±2.15	7.3±3.25	**6.45×10^{-13}**	5.71	9.97×10^{-1}	63.36	**2.77×10^{-4}**
TG 16:0/16:0/18:2	5,36±2.63	5.27±2.44	11.51±6.84	19.98±9.02	**1.43×10^{-12}**	−1.67	1.00×10^{0}	73.60	**3.01×10^{-4}**

TG									
16:1/16:1/18:0 TG	0.18±0.14	0.18±0.11	0.50±0.43	0.83±0.56	**3.26×10^{-10}**	-4.04	9.99×10^{-1}	67.28	**4.62×10^{-2}**
16:0/16:1/18:1 TG	8.52±5.39	8.25±4.4	19.97±9.51	32.03±16.98	**3.22×10^{-12}**	-3.16	1.00×10^{0}	60.43	**1.21×10^{-2}**
14:0/18:0/18:1 TG	0.12±0.10	0.12±0.09	0.34±0.33	0.69±0.63	**2.27×10^{-9}**	0.26	1.00×10^{0}	100.68	**3.68×10^{-2}**
16:0/16:0/18:1 TG	11.88±7.23	11.3±6.01	25.75±16.85	51.6±30.28	**4.34×10^{-12}**	-4.89	1.00×10^{0}	100.42	**5.77×10^{-4}**
16:0/16:0/18:0 TG	0.90±0.92	0.83±0.62	2.32±2.95	6.67±6.31	**6.79×10^{-10}**	-8.78	1.00×10^{0}	186.99	**2.67×10^{-3}**
15:0/18:1/18:1 TG	0.21±0.12	0.19±0.11	0.45±0.21	0.50±0.25	**5.85×10^{-10}**	-7.66	1.00×10^{0}	11.75	9.79×10^{-1}
16:1/17:0/18:1 TG	0.97±0.56	0.98±0.52	2.17±1.01	2.98±1.94	**8.71×10^{-11}**	0.86	1.00×10^{0}	37.26	2.65×10^{-1}
16:0/17:0/18:2 TG	0.71±0.38	0.81±0.49	1.53±0.83	2.68±1.83	**3.37×10^{-11}**	14.61	9.91×10^{-1}	75.71	**2.48×10^{-3}**
16:0/17:0/18:1 TG	0.37±0.22	0.43±0.33	0.86±0.58	1.64±1.55	**8.92×10^{-10}**	15.68	9.99×10^{-1}	91.49	**7.64×10^{-2}**
16:0/17:0/18:0 TG	0.04±0.03	0.04±0.04	0.08±0.10	0.27±0.42	**2.84×10^{-7}**	9.49	9.97×10^{-1}	222.10	**1.14×10^{-2}**
16:0/18:2/18:2 TG	12.53±4.18	13.09±5.41	27.78±10.5	33.31±9.92	**1.47×10^{-12}**	4.43	9.99×10^{-1}	19.88	2.07×10^{-1}
16:1/18:1/18:2 TG	6.66±2.56	6.97±3.05	15.96±4.89	18.37±5.42	**6.45×10^{-13}**	4.61	1.00×10^{0}	15.15	6.69×10^{-1}
16:1/18:1/18:1	2.12±1.11	2.26±1.10	5.24±1.67	6.00±2.17	**4.34×10^{-12}**	6.53	1.00×10^{0}	14.51	9.03×10^{-1}

TG									
16:0/18:1/18:2 TG	35.22±11.85	37.1±14.83	75.58±24.99	91.92±25.31	**6.45×10^{-13}**	5.34	1.00×10^{0}	21.62	7.96×10^{-2}
16:0/18:1/18:1 TG	34.19±14.87	36.08±16.53	73.52±28.56	100.79±38.74	**1.32×10^{-12}**	5.54	1.00×10^{0}	37.09	**4.62×10^{-2}**
16:0/18:0/18:1 TG	2.77±1.96	3.14±1.98	7.39±7.05	15.77±12.86	**3.73×10^{-10}**	13.34	5.30×10^{-1}	113.44	**2.12×10^{-2}**
17:0/18:1/18:1 TG	0.85±0.37	0.90±0.42	1.83±0.70	2.40±1.07	**1.33×10^{-11}**	6.59	7.20×10^{-1}	31.12	1.98×10^{-1}
18:2/18:2/18:2 TG	0.95±0.38	0.99±0.47	2.25±1.16	2.03±0.83	**4.26×10^{-9}**	3.73	7.80×10^{-1}	−9.71	9.91×10^{-1}
18:1/18:2/18:2 TG	5.38±1.74	5.51±2.12	11.83±5.17	11.27±3.74	**2.95×10^{-11}**	2.44	8.20×10^{-1}	−4.71	1.00×10^{0}
18:0/18:2/18:2 TG	0.88±0.34	0.98±0.37	1.79±0.86	1.99±0.64	**1.14×10^{-10}**	11.96	2.70×10^{-1}	10.97	6.69×10^{-1}
18:1/18:1/18:2 TG	7.41±2.46	7.82±2.8	15.93±6.18	15.42±4.64	**8.32×10^{-12}**	5.60	6.40×10^{-1}	−3.22	1.00×10^{0}
18:1/18:1/18:1 TG	5.33±2.21	5.75±2.25	11.08±4.21	11.94±4.42	**4.65×10^{-11}**	8.01	5.20×10^{-1}	7.74	8.95×10^{-1}
18:0/18:1/18:1 TG	2.03±0.95	2.44±1.29	4.95±3.32	7.63±5.29	**1.40×10^{-10}**	20.52	2.80×10^{-1}	54.12	7.17×10^{-2}
18:0/18:0/18:1 TG	0.21±0.11	0.26±0.18	0.58±0.6	1.28±1.45	**3.42×10^{-9}**	26.00	4.70×10^{-1}	122.76	**1.78×10^{-2}**
18:0/18:0/18:0 TG	0.01±0.01	0.01±0.01	0.03±0.03	0.07±0.12	**2.56×10^{-7}**	5.05	9.90×10^{-1}	169.25	**1.07×10^{-2}**
18:2/18:2/20:4	0.06±0.01	0.07±0.02	0.12±0.05	0.12±0.04	**3.49×10^{-10}**	12.17	1.40×10^{-1}	−3.45	1.00×10^{0}

TG 18:1/18:1/20:4	0.12±0.05	0.13±0.05	0.24±0.10	0.26±0.09	**6.47×10^{-11}**	3.64	8.70×10^{0}	7.78	5.96×10^{-1}
TG 18:1/18:1/22:6	0.12±0.09	0.15±0.09	0.17±0.08	0.20±0.08	**1.04×10^{-4}**	26.39	**2.45×10^{-1}**	15.24	8.67×10^{-1}

a All values are presented as the means±SD of plasma lipid concentration in umol/L.

b Percentage (%) of difference between obese and normal-weight adolescents.

c We performed Benjamini-Hochberg correction for multiple testing.

d The black font was statistically significant.

e Abbreviations: NN, Normal-weight with traditional Normal clinical plasma lipid levels; NA, Normal-weight with traditional Abnormal clinical plasma lipid levels; ON, Obese with traditional Normal clinical plasma lipid levels; OA, Obese with traditional Abnormal clinical plasma lipid levels
